# Aminosteroid RM-581 Induces G0/G1 Arrest and Endoplasmic Reticulum Stress-Mediated Apoptosis in Human Acute and Chronic Leukemia Cell Lines

**DOI:** 10.3390/cancers18071078

**Published:** 2026-03-26

**Authors:** Maude Fleury, Jenny Roy, René Maltais, Francine Durocher, Donald Poirier

**Affiliations:** 1 Endocrinology and Nephrology Unit, CHU de Québec-Université Laval Research Center, Pavillon CHUL, Québec, QC G1V 4G2, Canada; maude.fleury@crchudequebec.ulaval.ca (M.F.); jenny.roy@crchudequebec.ulaval.ca (J.R.); rene.maltais@crchudequebec.ulaval.ca (R.M.); francine.durocher@crchudequebec.ulaval.ca (F.D.); 2 Department of Molecular Medicine, Faculty of Medicine, Université Laval, Québec, QC G1V 0A6, Canada; 3 Université Laval Cancer Research Center, Québec, QC G1R 3S3, Canada

**Keywords:** leukemia, steroid, endoplasmic reticulum stress, apoptosis, cell cycle

## Abstract

Background: The aminosteroid RM-581 exhibits strong antiproliferative activity against cell lines from more than 10 solid tumor cancers, including some with poor prognoses. However, RM-581’s impact has never been assessed on leukemia. Methods: Cellular responses to RM-581 were evaluated using complementary approaches. Cytotoxicity was quantified using MTS-based viability assays and drug interactions were analyzed according to the Chou-Talalay method. Flow cytometry was employed to assess apoptosis, cell cycle distribution and effects on lymphocytes subpopulations. The transcriptomic profile was investigated by mRNA sequencing to identify differentially expressed genes and associated pathways. Results: Its evaluation on six leukemia cell lines (HL-60, THP-1, JURKAT, K-562, HG-3 and JVM-2) showed that RM-581 efficiently blocked the proliferation of leukemia cells. In healthy peripheral blood lymphocytes, flow cytometry revealed a significant impact on T lymphocytes (CD3+), particularly cytotoxic T cells (CD8+), at 50 µM. In THP-1 cells, an acute monocytic leukemia cell line, RM-581 triggered apoptosis and induced G0/G1 cell cycle arrest, which was confirmed with a transcriptomic analysis of enriched pathways. The role of RM-581 as an endoplasmic reticulum (ER) stress aggravator was confirmed by observing an increase in ER stress markers, such as BIP (GRP-78), CHOP and HERP, and in unfolded protein response (UPR) effectors (PERK, IRE1α and ATF6). Conclusions: This study demonstrates that RM-581 could be a promising candidate to treat leukemia, notably through the induction of ER-stress mediated apoptosis.

## Simple Summary

Leukemia remains the 10th most lethal cancer across all age groups. Numerous treatments are available, but with major adverse effects. A novel anticancer agent, the aminosteroid derivative RM-581, has promising antiproliferative activity against various solid tumors in vitro and in vivo, including some cancers with poor prognosis. The aim of this study was to determine its potential as a treatment against leukemia. It was confirmed that RM-581 acted as a pro-apoptotic agent that induced endoplasmic reticulum stress in leukemia cell lines, the same as in solid tumor cell lines. The most interesting result was its ability to induce G0/G1 arrest in THP-1 cells as early as 24 h post-treatment, which was confirmed by the mRNA expression of the proteins implicated in the G0/G1 phase. These findings provided valuable insight into RM-581’s mechanism of action as a promising anticancer drug.

## 1. Introduction

While leukemia is the most frequent childhood cancer, with an incidence rate of 35% and a mortality rate of 23% in young Canadians between the ages of 0 and 14, it remains a significant health concern among adults [1]. The International Agency for Research on Cancer (IARC) estimated that, in 2022, approximately 490,000 new cases of leukemia were diagnosed, and 305,000 deaths were reported worldwide, making it the 10th most lethal cancer across all ages [2]. Leukemia is a heterogeneous group of hematological malignancies caused by the uncontrolled proliferation of abnormal white blood cells, which impairs normal bone marrow function. Leukemia can be classified into four major types, based on the cell lineage (myeloid or lymphocytic) and the degree of cell maturity prior to carcinogenic transformation (acute or chronic): acute myeloid leukemia (AML), chronic myeloid leukemia (CML), acute lymphoblastic leukemia (ALL) and chronic lymphocytic leukemia (CLL) [3,4,5]. Each leukemia subtype has a different prevalence, prognosis and treatment, based on the molecular profile and patient factors (age, comorbid conditions, and risk factors) [3], making leukemias difficult to treat, due to genetic variability, metabolic diversity, and heterogeneity across patients [5]. Although numerous chemotherapeutic agents are available, the treatment of leukemia often requires the administration of multiple high doses of chemotherapy [6]. These challenges highlight the need for novel therapeutic agents working through a different mechanism of action than those already used, which can treat leukemia more effectively.

Chemotherapy is the main course of leukemia treatment, but depending on the type, non-chemo drugs like tyrosine kinase inhibitors, all-trans-retinoic acid (ATRA) and corticosteroids can also be used in the clinic [3,7]. While corticosteroids (prednisone and dexamethasone) are well established in leukemia therapy, investigations into other steroidal compounds remain relatively limited [7,8,9]. Over the last few years, various aminosteroid derivatives were designed, synthesized, and evaluated by our research group as anticancer agents [10,11,12]. They represent a new family of pro-apoptotic molecules that have high cytotoxic effects on multiple types of cancer, both in vitro and in vivo [12,13,14,15,16]. Among these, the aminosteroid RM-581 was the most successful candidate to date. It demonstrated antiproliferative activity against various cancer cell lines, including some cancers with poor prognosis [10,13,17]. Although its molecular target has yet to be identified, RM-581 induces endoplasmic reticulum (ER) stress, leading to apoptosis in multiple cancer cell lines (breast, pancreas, and prostate) [12,16,17]. When administered orally, it also effectively inhibited tumor growth in nude mouse xenograft models of pancreas (PANC-1), prostate (LAPC-4 and PC-3) and breast (MCF-7) cancers, and no apparent signs of toxicity were observed in treated nude mice (3–60 mg/kg) as well as in normal mice at a dose as high as 720 mg/kg [10,16,17,18].

Considering the promising results obtained with RM-581 on solid tumors, we decided to extend its evaluation to leukemia cancer models. To this end, we tested RM-581’s antiproliferative effects on leukemia cell lines belonging to the four subcategories of leukemia (AML, ALL, CML and CLL), alone and in combination with doxorubicin, a standard chemotherapeutic agent used in the clinic against ALL and AML. We also assessed its impact on normal cells (lymphocytes). We further investigated its molecular mechanism by performing apoptosis characterization, cell cycle analysis, and mRNA-seq-base differential gene expression and functional enrichment analyses.

## 2. Materials and Methods

### 2.1. Aminosteroid RM-581

Aminosteroid RM-581 was prepared as previously reported, and its purity was 99.6%, as determined by high-performance liquid chromatography [19].

### 2.2. Cell Lines

Human leukemia HL-60 (AML), THP-1 (AML), JURKAT (ALL), K562 (CML), HG-3 (CLL) and JVM-2 (CLL) cells were obtained from the American Type Culture Collection (ATCC, Rockville, MD, USA). All leukemia cell lines were grown in RPMI-1640 media supplemented with 10% fetal bovine serum (FBS), antibiotics (100 IU penicillin/mL and 100 mg streptomycin/mL) and L-glutamine (2 nM). For CLL cell lines, however, the medium was supplemented with sodium pyruvate (1%). Normal peripheral blood lymphocytes (PBLs) were isolated from normal peripheral blood mononuclear cells (PBMCs) purchased from BioIVT (Westbury, NY, USA) in two independent lots (Lot #HMN359229 and Lot #HMN697339). PBLs were grown in RPMI-1640 media supplemented with 10% fetal bovine serum (FBS), antibiotics (100 IU penicillin/mL and 100 mg streptomycin/mL), L-glutamine (2 nM) and phytohemagglutinin-L (PHA-L) (2 µg/mL) (Sigma-Aldrich, Oakville, ON, Canada). All the cell lines were incubated at 37 °C with 5% CO_2_ in a water-saturated atmosphere. Cells were split once a week to maintain cell propagation.

### 2.3. Cell Viability Assays and Combination Index Assays

Cells were seeded in 96-well plates in triplicate (1 × 10^4^ cells/well) in culture medium. The cells were incubated at 37 °C in a humidified atmosphere for 24 h before treatment. RM-581 was diluted in dimethylsulfoxide (DMSO) to prepare the stock solution (10^−2^ M) and added at increasing concentrations (0.05, 0.1, 1, 5, 20 and 50 µM) in the culture medium. The control cells were treated with the culture medium containing 0.2% of DMSO. For combination index (CI) assays, cells received a RM-581 and doxorubicin treatment at the same concentrations (0.05, 0.1, 1, 3, 5 or 20 µM). After 3 days of incubation, 20 µL of 3-(4,5-dimethylthiazol-2-yl)-5-(3-carboxymethoxyphenyl)-2-(4-sulfophenyl)-2H-tetrazolium (MTS) was added to each well, and the mixture was incubated for 2–6 h. The plates were then analyzed at 490 nm using a Tecan M-200 microplate reader (Männedorf, Switzerland). Percentage viability was calculated for the treated cells compared with the untreated cells receiving only DMSO (0.2%) in the culture medium. For the cell viability assays, the IC_50_ (50% inhibitory concentration of cell proliferation) values were calculated using the GraphPad Prism 7 software. The values represent the average of three to five experiments performed in triplicate (mean ± SD).

For the *CI* determination, according to the Chou–Talalay method [20], the following formula was used:

(1) $$CI=\;\frac{{\left(D\right)}_{1}}{{\left(D_{X}\right)}_{1}}+\frac{{\left(D\right)}_{2}}{{\left(D_{X}\right)}_{2}}$$

Denominators (*DX*)_1_ and (*DX*)_2_ are the doses of individual drugs required to achieve a given effect level, and numerators (*D*)_1_ and (*D*)_2_ are the concentrations of each drug present in combination to trigger the same effect level. A *CI* = 1 indicates an additive interaction, a *CI* < 1 indicates a synergistic interaction, and a *CI* > 1 indicates an antagonistic effect.

### 2.4. Flow Cytometric Analysis of Cell Surface Antigens on PBLs

PBLs were seeded for 48 h in 96-well plates (1 × 10^5^ cells/well) in culture medium (90 µL) supplemented with PHA-L. RM-581 was diluted in DMSO to prepare the stock solution (10^−2^ M) and added at increasing concentrations (0.1, 1, 5, 20 and 50 µM) in the culture medium. Normal PBLs without treatment with RM-581 were used as a control to determine the basal level of expression of cell surface antigens. The control cells were treated with the culture medium containing 0.2% of DMSO. After 72 h of incubation, the cells were harvested, washed with 1 mL of cold phosphate-buffered saline (PBS), centrifuged for 5 min at 350× *g*, and resuspended in 100 µL of PBS. The cells were stained first with Ghost Dye Red 780 (0.05 µL per 1 × 10^5^ cells) (Cytek Biosciences Inc., Fremont, CA, USA) according to the manufacturer’s instructions. The cells were then washed and stained with anti-CD19-FITC (2.5 µL per 1 × 10^5^ cells), anti-CD3-PE (2.5 µL per 1 × 10^5^ cells), anti-CD4-PE/Cy7 (1 µL per 1 × 10^5^ cells) and anti-CD8-PerCP (1 µL per 1 × 10^5^ cells) (Biolegend Inc., San Diego, CA, USA) and incubated on ice for 20 min in the dark. After the incubation period, the cells were washed with 1 mL of PBS, centrifuged for 5 min at 350× *g* and resuspended in 300 µL of PBS. Finally, the cells were analyzed by flow cytometry using a Northern Lights flow cytometer (Cytek Biosciences Inc., Fremont, CA, USA) on the Flow Cytometry Platform (CHU de Québec-Université Laval Research Center, Quebec City, QC, Canada).

### 2.5. Apoptosis Characterization by Flow Cytometry

THP-1 cells were plated at a density of 2 × 10^5^ cells per well in 6-well plates and allowed to stabilize overnight in 3 mL of complete culture medium. The cells were subsequently treated with RM-581 at concentrations of 2, 10, 30 and 50 µM for a period of 3 days. Following treatment, the cells were harvested by centrifugation and washed with ice-cold PBS. The collected cells were resuspended and incubated for 15 min in the dark at room temperature in 200 µL of annexin-binding buffer containing 2.5 µL of annexin-V Pacific Blue and 1 µL of propidium iodide (PI) at 0.1 mg/mL. After staining, an additional 500 µL of annexin-binding buffer was added prior to immediate acquisition with a Northern Lights flow cytometer (Cytek Biosciences Inc., Fremont, CA, USA) on the CHU de Québec–Université Laval flow cytometry platform. Cell populations were classified based on annexin V and PI staining profiles: viable cells (annexin V^−^/PI^−^), early apoptotic cells (annexin V^+^/PI^−^), late apoptotic cells (annexin V^+^/PI^+^), and necrotic cells (annexin V^−^/PI^+^).

### 2.6. Cell Cycle Analysis by Flow Cytometry

THP-1 cells (5 × 10^5^ cells per well) were seeded in 6-well plates and incubated overnight prior to treatment. The cells were then exposed to RM-581 (10 µM) for 24, 48 or 72 h. Control conditions consisted of cells treated with culture medium containing DMSO (0.2%). At each time point, cells were collected by centrifugation (300 g, 5 min, and 4 °C), washed with PBS, and fixed by the addition of cold ethanol. The fixed samples were maintained at −20 °C for a minimum of 30 min prior to further processing. Following fixation, the cells were pelleted and resuspended in PBS containing RNase A (500 IU/mL) and then incubated at 37 °C for 30 min. DNA content was subsequently stained using PI (1 mg/mL), and the samples were incubated on ice in the dark for 20 min. Flow cytometric analysis was performed using a Northern Lights instrument (Cytek Biosciences Inc., Freemont, CA, USA), and the cell cycle distribution (G0/G1, S and G2/M phases) was determined using the FCS Express software v7.26 (De Novo Software, Pasadena, CA, USA).

### 2.7. Gene Expression Study by mRNAseq

THP-1 cells were seeded at 3 × 10^5^ cells per well and treated with RM-581 (10 µM) for 6, 12 or 24 h. Total RNA was extracted using QIAzol reagent followed by purification with the miRNeasy Micro Kit, including on-column DNase digestion, according to the manufacturer’s protocol (Qiagen, Hilden, DE, USA). The RNA concentration was determined using a NanoDrop spectrophotometer (NanoDrop Technologies, Wilminton, DE, USA), while RNA integrity was assessed with an Agilent TapeStation 4150 system using High-Sensitivity RNA ScreenTape (Agilent Technologies, Santa Clara, CA, USA).

For transcriptomic profiling, mRNA libraries were generated using the NEBNext Ultra II Directional RNA Library Preparation Kit (New England Biolabs, Ipswich, MA, USA). Polyadenylated transcripts were first isolated and then reverse-transcribed into cDNA using random primers. Strand specificity was ensured by incorporating dUTP during second-strand synthesis. After end repair and adapter ligation, the libraries were purified, subjected to limited PCR amplification (9 cycles), and indexed for multiplexing. Library quality and size distribution were verified using DNA ScreenTape D1000 on a TapeStation 2200) Agilent Technologies, Santa Clara, CA, USA), and quantification was performed with a Qubit fluorometer (ThermoFisher Scientific, Waltham, MA, USA). Equimolar pooling of indexed libraries was followed by paired-end sequencing (2 × 100 bp) on an Illumina NovaSeq 6000 at the CHU de Québec–Université Laval Genomics Center, generating an average of ~19 million reads per sample. Raw sequencing reads were processed using fastp (v0.23.2) [21] for trimming. Quality metrics were evaluated with FastQC (v0.11.9) [22] and aggregated using MultiQC (v1.12) [23]. Transcript abundance was estimated using Kallisto (v0.48.0) [24] against the GRCh38 human transcriptome (Ensembl release 110). The principal component analysis (PCA) was completed with the FactoMineR v2.9 R package [25]. The PCA and volcano graphical representations were produced with the ggplot2 v3.4.4 package [26]. Differential expression analysis was also performed using the DESeq2 v1.40.2 package [27]. All R analyses were carried out in R v4.3.1 [28].

### 2.8. Statistical Analysis

Statistical analyses were conducted using GraphPad Prism (version 10.6.1, GraphPad Software, Boston, MA, USA). Comparisons between multiple groups were performed using two-way ANOVA followed by Dunnet’s test, whereas other comparisons were evaluated using Student’s *t*-test when appropriate. A *p*-value of < 0.05 was considered indicative of statistical significance.

## 3. Results

### 3.1. Antiproliferative Activity of RM-581 Against Leukemia Cell Lines

We assessed the antiproliferative activity of RM-581 against six leukemia cell lines with a range of concentrations to determine the IC_50_. As shown in Table 1, the IC_50_ values of RM-581 for these cell lines were: 1.71 µM for HL-60, 5.61 µM for THP-1, 1.18 µM for JURKAT, 4.47 µM for K-562, 2.49 µM for HG-3 and 2.71 µM for JVM-2. Although HL-60 and JURKAT cells exhibited better sensitivity to RM-581, THP-1 cells were selected for additional studies due to their relevance as an acute monocytic leukemia model and their use in studies of therapeutic responses [29].

**Figure 1:**
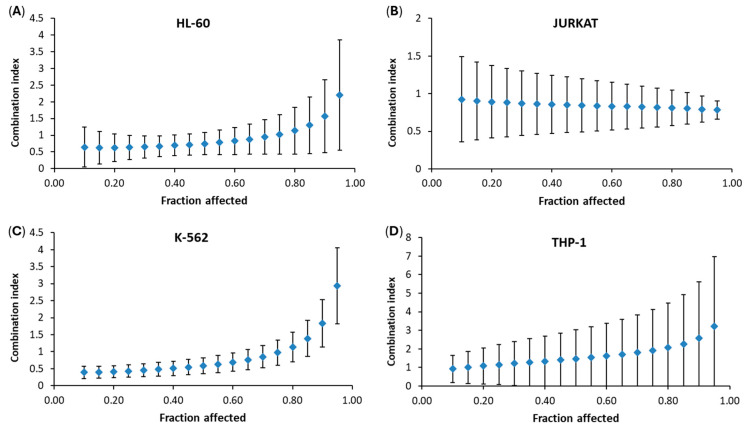
Combination index (*CI*) of RM-581 with doxorubicin using the Chou–Talalay method in (**A**) HL-60 cells, (**B**) JURKAT cells, (**C**) K-562 cells and (**D**) THP-1 cells. Drug synergy, addition and antagonism are defined by *CI* values less than 1.0, equal to 1.0, or greater than 1.0, respectively.

As shown in Table 1, we evaluated the activity of doxorubicin, a standard chemotherapeutic agent, on all leukemia cell lines, where its IC_50_ values ranged from 0.017 to 0.88 µM. Doxorubicin exerts its cytotoxicity via the inhibition of topoisomerase II, the generation of reactive oxygen species and the direct stimulation of apoptosis [30]. The Chou–Talalay method [20] was applied to evaluate the interactions between RM-581 and doxorubicin in four leukemia cell lines. Combination effects varied across the fraction affected (Fa), representing the proportion of cells inhibited by the combined treatment. HL-60 and K-562 cells exhibited synergy at low Fa (<0.50) and antagonism at high Fa (>0.50) (Figure 1A,C), whereas THP-1 cells displayed a shift from additivity to antagonism (Figure 1D). In contrast, JURKAT cells demonstrated a sustained additive effect at all Fa levels (Figure 1B). These data indicate that the RM-581/doxorubicin interaction was cell-line dependent and changed across the Fa range.

### 3.2. Subpopulations of PBLs Affected by RM-581

Flow cytometry analysis of PBL cell surface antigens was performed on the remaining viable single cells (Table S1A) to determine the effect of RM-581 on the proliferation of different PBL populations. The antigens analyzed were CD3 (T-lymphocytes), and its subpopulations CD4 (T helper) and CD8 (T cytotoxic), and CD19 (B-lymphocytes). The samples were analyzed according to % [CD19-positive] (B cells) + % [CD3-positive] (T cells) + % [CD19-negative + CD3-negative] ≈ 100% [31]. For this experiment with healthy PBLs, RM-581 exhibited an IC_50_ of 7.4 ± 3.6 µM (Table S1A), whereas in THP-1 cells, an IC_50_ of 14.9 ± 6.8 µM was observed under the same experimental conditions (Table S1B), suggesting no selectivity for cancer cells.

**Figure 2:**
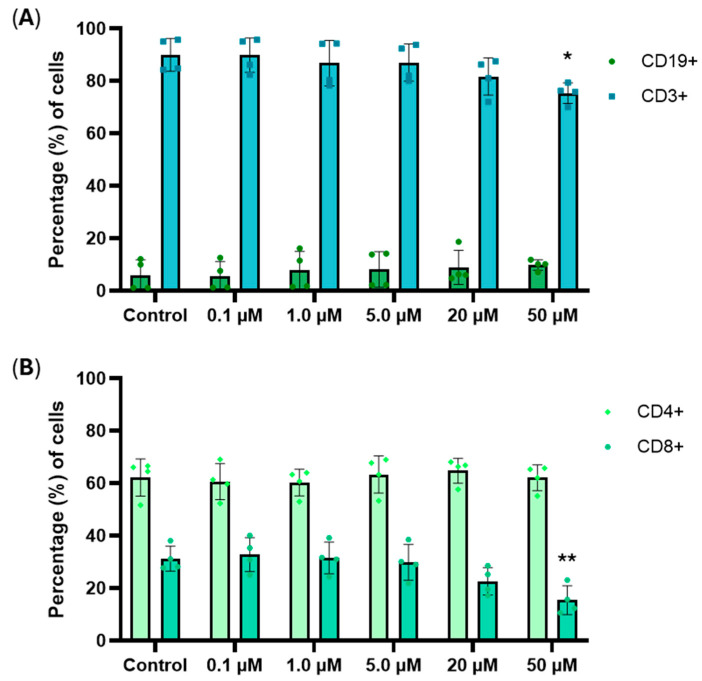
Analysis of the percentage (%) of subpopulations of normal PBLs (1 × 10^5^ cells/well) treated for 3 days with RM-581 and not (control). (**A**) Effect of RM-581 on the CD19+ (B cells) and CD3+ (T cells) populations. (**B**) Effect of RM-581 on T cells CD4+ (helper) and CD8+ (cytotoxic). Each data point represents the mean of four independent experiments (mean ± SD). Two-way ANOVA. * *p* < 0.05 and ** *p* < 0.005 versus control.

**Figure 3:**
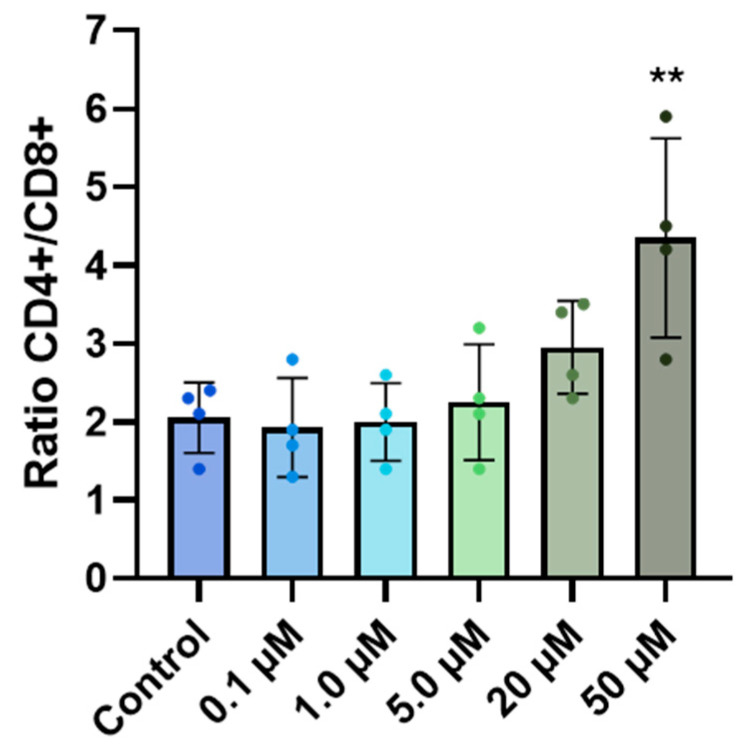
Effect of RM-581 on the ratio CD4+/CD8+ of PBLs (1 × 10^5^ cells/well) treated for 3 days. Each data point represents the mean of four independent experiments (mean ± SD). One-way ANOVA. ** *p* < 0.005 versus control.

A decrease in the percentage of T-lymphocytes (CD3+) was observed according to the concentration and was significant at 50 µM vs. control (75.4% and 89.9%, respectively), while the B-lymphocyte (CD19+) population showed a tendency to increase (6.1% to 9.9%) (Figure 2A). Similarly, the subpopulation of CD8+ T-lymphocytes decreased from 31.3% to 15.4% and was significant at 50 µM of RM-581, whereas no significant change was noted for the CD4+ T-lymphocyte subpopulation (Figure 2B). The CD4+/CD8+ ratio was significantly increased when treated with 50 µM of RM-581 (2.05 to 4.35) (Figure 3). The results obtained demonstrated that RM-581 affects T-lymphocytes (CD3+) more importantly than B-lymphocytes (CD19+), with the subpopulation of CD8+ T-lymphocytes being more sensitive.

### 3.3. Apoptosis Characterization in THP-1 Cells

**Figure 4:**
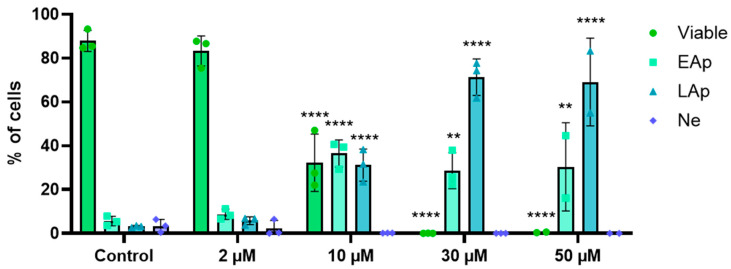
Impact of RM-581 on cell viability and cell death modalities in THP-1 cells. Cells (2 × 10^5^) were treated for 72 h with increasing concentrations of RM-581 (2–50 µM). Following treatment, apoptotic and necrotic populations were quantified by flow cytometry using annexin V and propidium iodide staining. Cell populations were classified as viable, early apoptotic (EAp), late apoptotic (LAp), or necrotic (Ne) based on staining profiles. Data are presented as the means ± SD from three independent experiments. Statistical analysis was performed using two-way ANOVA; significance levels are indicated as follows: ** *p* < 0.005 and **** *p* < 0.0001 compared with the control.

The effect of RM-581 on THP-1 cell death was assessed by flow cytometry using annexin V/PI staining, where THP-1 cells were treated for 72 h with increasing concentrations (2–50 µM). A concentration-dependent induction of apoptosis was observed (Figure 4). In untreated control cells, most of the population remained viable (87.8%), with low percentages of early apoptotic (5.6%), late apoptotic (3.1%), and necrotic (3.4%) cells. The lowest concentration (2 µM) did not significantly affect cell viability or apoptosis when compared with the control. At 10 µM, viability decreased to 32.2%, while early and late apoptotic cells significantly increased to 36.5% and 31.2%, respectively. Late apoptosis became predominant at higher concentrations (30–50 µM), with respective proportions of 71.3% and 69.1%. The proportion of necrotic cells remained low (<5%) and non-significant across all conditions, indicating that cell death was primarily apoptotic rather than necrotic. These data demonstrate that RM-581 induces apoptosis in THP-1 cells in a concentration-dependent manner, leading to a near-complete loss of viability at the highest concentrations. Apoptosis was also characterized in THP-1 cells treated with doxorubicin, the reference compound, which exhibited a comparable profile to that of RM-581 (Figure S1).

### 3.4. G0/G1 Cell Cycle Arrest in THP-1 Cells

**Figure 5:**
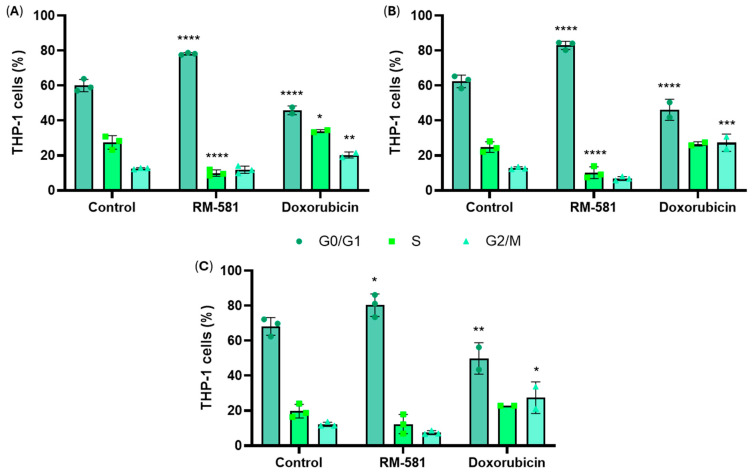
Cell cycle analysis of THP-1 cells (5 × 10^5^ in 3 mL) after treatment with 0.2% DMSO (control), 10.0 µM of RM-581 or 0.1 µM of doxorubicin. After (**A**) 24 h, (**B**) 48 h and (**C**) 72 h of treatment, cells were labeled with propidium iodide dye and analyzed by flow cytometry. Each data point represents the mean of 3 independent experiments (mean ± SD). Two-way ANOVA. * *p* < 0.05, ** *p* < 0.005, *** *p* < 0.001, and **** *p* < 0.0001 versus control.

The influence of RM-581 and doxorubicin on cell cycle changes in THP-1 cells was evaluated by performing cell-cycle analysis of cells treated in a time-course manner with 10 µM of RM-581 and 0.1 µM of doxorubicin for comparison. After 24 h, a significant increase in the proportion of cells treated with RM-581 in the G0/G1 phase was observed (78.2% versus 59.9% in the control) (Figure 5A). A significant accumulation of THP-1 cells in the G0/G1 phase was also present after 48 h and 72 h (83.0% and 80.3%, respectively) of treatment with RM-581 (Figure 5B,C). Correspondingly, in cells treated with RM-581, the S-phase population was significantly reduced at 24 h and 48 h (10.0% and 10.2%, respectively), while the G2/M cell distribution remained unchanged. Thus, these results suggest that RM-581 slows down the proliferation of THP-1 cells by inducing a G0/G1 cell cycle block. In contrast with RM-581, doxorubicin caused a significant decrease in the G0/G1 population and a significant accumulation of cells in the G2/M phase across all time points. A transient increase in S-phase cells was also observed at 24 h (33.9%).

**Figure 6:**
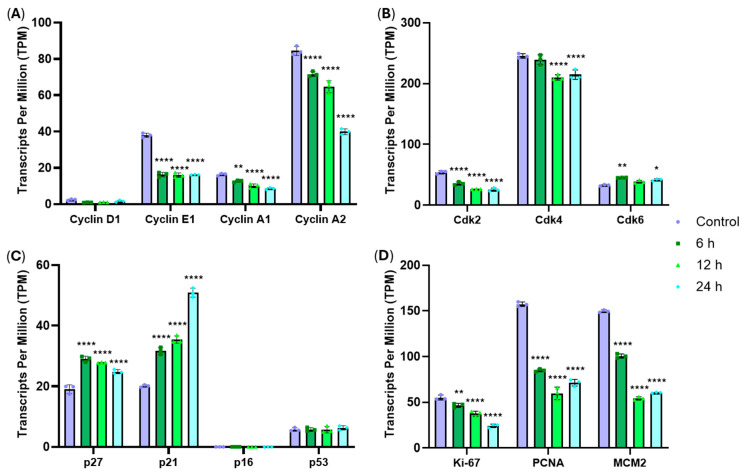
Cell cycle G0/G1 phase-related gene expression profiles, such as (**A**) cyclins, (**B**) CDKs, (**C**) CKIs and (**D**) proliferation markers in THP-1 cells (3 × 10^5^) exposed for 6 h, 12 h and 24 h to 10 µM of RM-581, analyzed by mRNA sequencing and normalized by transcript per million (TPM). Each data point represents the mean of an experiment performed in triplicate (mean ± SD). Two-way ANOVA. * *p* < 0.05, ** *p* < 0.005 and **** *p* < 0.0001 versus control.

To investigate the molecular events that caused G0/G1 arrest in cells treated with RM-581, the expression of cell cycle regulatory genes involved in G1 progression and G1/S transition was evaluated by RNA sequencing. THP-1 cells were exposed to 10 µM of RM-581 in a time-course manner (6, 12 and 24 h). Across all time points, the expression levels of cyclin E1 and cyclin A (*CCNA1/2*), two cyclins that promote progression through G1/S phases, as well as CDK2 and CDK4, two cyclin-dependent kinases (CDKs), were decreased compared with the control (Figure 6A,B). In contrast, the expression of p27 and p21, two cyclin-dependent kinase inhibitors (CKIs), was increased (Figure 6C). Notably, CDK6 showed elevated expression specifically at 6 and 24 h. Consistent with reduced proliferative activity, the proliferation markers Ki-67, PCNA and MCM2 also displayed decreased expression (Figure 6D). Additional genes implicated in the MCM protein complex (MCM2-7) were also downregulated (Figure S2). Finally, no significant changes were observed in the expression of cyclin D1, p16 and p53. This transcriptional profile is consistent with a G0/G1 arrest that prevents S-phase entry.

### 3.5. Transcriptomic Changes Induced by RM-581 in THP-1 Cells

**Figure 7:**
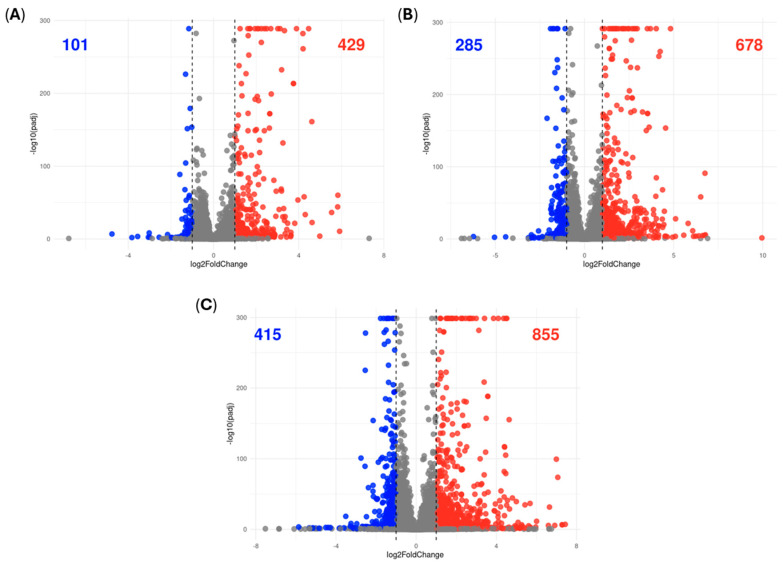
Volcano plot of differentially expressed genes (DEGs) using an absolute fold change threshold of 2 (padj ≤ 0.05) for the (**A**) 6 h, (**B**) 12 h and (**C**) 24 h versus control. Each dot represents a DEG, where the blue dots represent downregulated genes, the red dots represent the upregulated genes, and the gray dots represent non-significant genes. Each data point represents the mean of an experiment performed in triplicate.

We used RNA-seq analysis to determine the effect of a time-course treatment with RM-581 (10 µM) on the transcriptomic profile of THP-1 cells. A progressive increase in differentially expressed genes (DEGs) was observed in levels of more than two-fold with significant *p*-values (≤0.05) (Figure 7; Supplementary Tables S2–S4). At each time point, there were double the upregulated DEGs (red dots) compared with the downregulated DEGs (blue dots), going from 429 to 101 DEGs at 6 h (Figure 7A), 678 to 285 DEGs at 12 h (Figure 7B) and 855 to 415 DEGs at 24 h (Figure 7C). A heatmap analysis was done with DEGs of interest representing approximately 10 functional categories, such as cell signaling, cholesterol biosynthesis, chromatin, death receptors, lipid biosynthesis, mitochondrial and energy metabolism, nuclear receptors, proteases, transcription factors, unfolded protein response pathway and others, which highlights change within categories over time (Figure S3).

**Figure 8:**
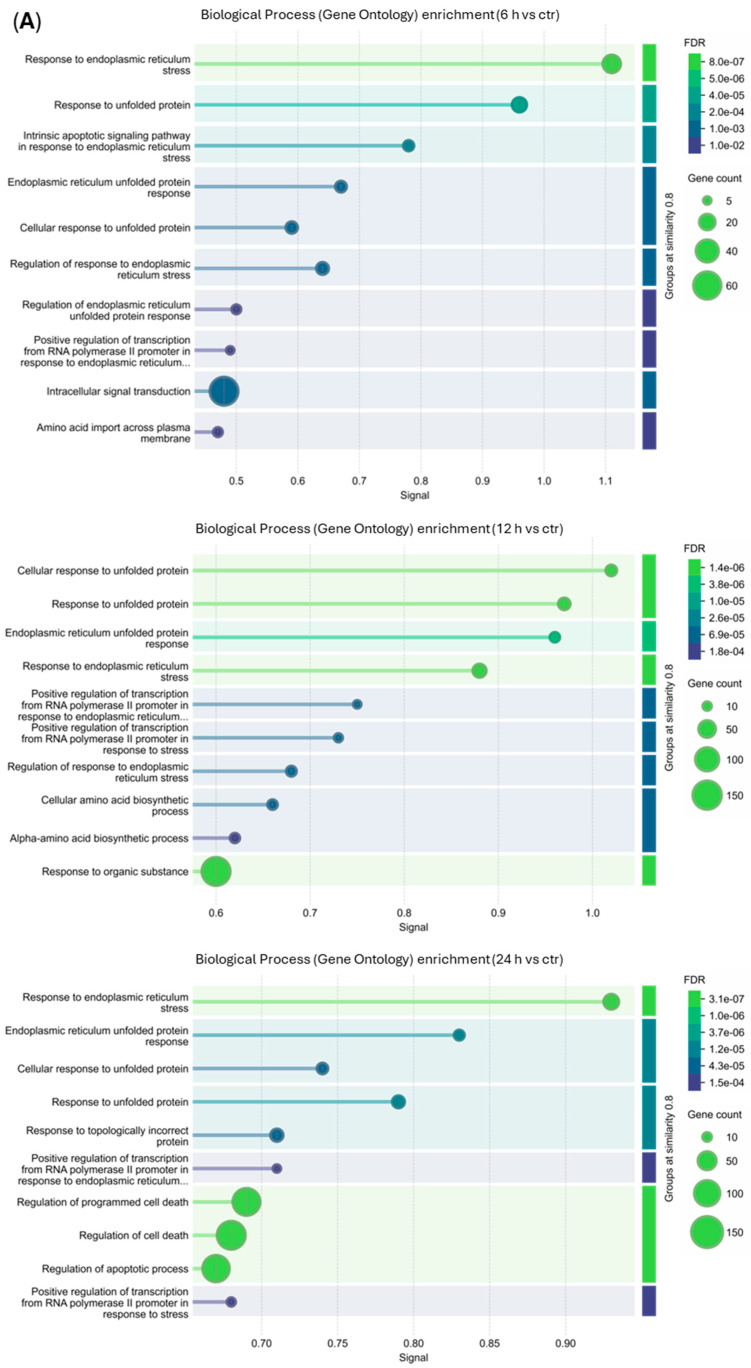

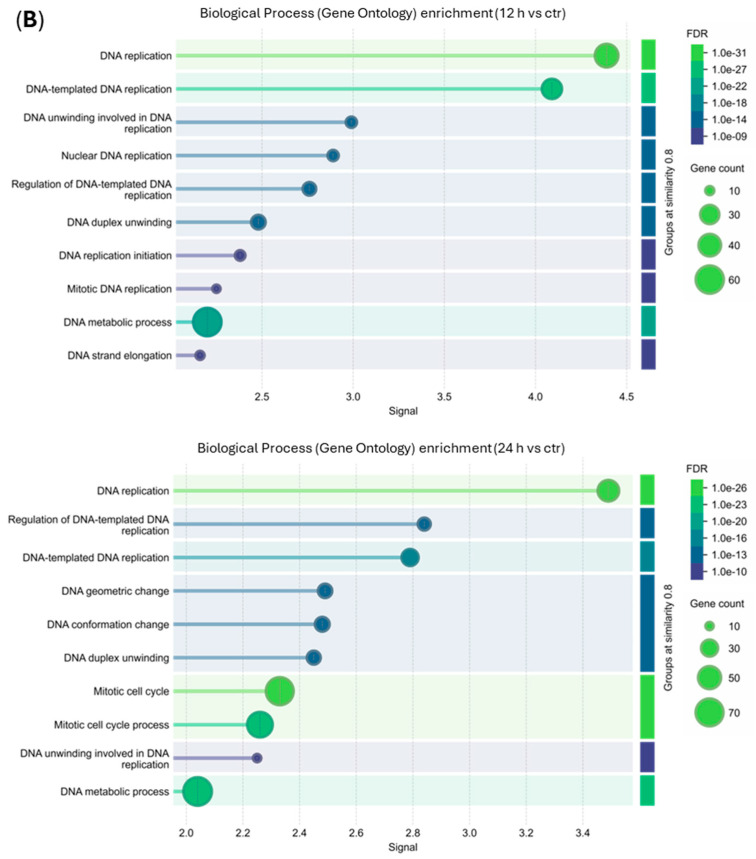
Biological process (Gene Ontology) enrichment analysis of (**A**) upregulated genes (6, 12 and 24 h) and (**B**) downregulated genes (12 and 24 h) in THP-1 cells (3 × 10^5^) treated with RM-581 (10 µM). Enrichment analysis was performed using STRING v12 (https://string-db.org/, accessed on 14 October 2025) [32]. Only terms with FDR-adjusted *p* ≤ 0.05 are displayed. Adapted from the original STRING output by renaming the titles to match experimental groups.

To determine the biological processes (BPs) activated in THP-1-treated cells, enrichment analysis was performed by transferring the lists of upregulated or downregulated genes at each time point into the STRING software v12 [32]. The enrichment analysis done with upregulated DEGs revealed that 67, 107 and 155 BPs were enriched at 6, 12 and 24 h, respectively (Supplementary Tables S5–S7), while the enrichment analysis done with downregulated DEGs showed that 109 and 157 BPs were enriched at 12 and 24 h, respectively (Supplementary Tables S8 and S9). The analysis results of the top 10-degree values in each condition were selected and presented as bubble diagrams (Figure 8).

The upregulated DEGs presented similar top 10 enriched BPs at the three timepoints, such as response to ER stress, response to unfolded protein, ER unfolded protein response, cellular response to unfolded protein and positive regulation of transcription from RNA polymerase II promoter in response to ER stress. At 24 h, there was an enrichment of the upregulated DEGs participating in the regulation of cell death and of the apoptosis process (Figure 8A).

The downregulated DEGs showed no enriched BPs at 6 h post-treatment. However, at 12 h and 24 h, similar top 10 BPs were enriched, like DNA replication, DNA-templated DNA replication, DNA unwinding involved in DNA replication, regulation of DNA-templated DNA replication, DNA duplex unwinding and DNA metabolic process (Figure 8B).

**Figure 9:**
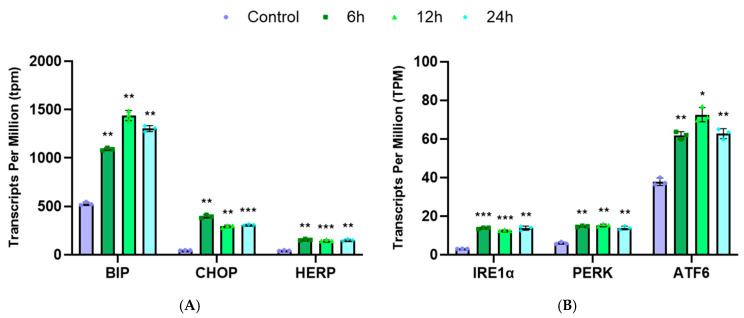
RNA-seq analysis of (**A**) ER stress markers (BIP, CHOP and HERP) and (**B**) of unfolded protein response factors (IRE1α, PERK and ATF6) in RM-581-treated THP-1 cells (3 × 10^5^). Each data point represents the mean of an experiment performed in triplicate (mean ± SD). Two-way ANOVA. * *p* < 0.05, ** *p* < 0.005, and *** *p* < 0.001 versus control.

The enrichment analysis showed that the upregulated genes were mainly implicated in the ER unfolded protein response. Thus, the mRNA expression levels of ER stress markers (BIP, CHOP and HERP) and ER transmembrane proteins (inositol-requiring enzyme 1 alpha (IRE1α), protein kinase RNA-like ER kinase (PERK) and activating transcription factor 6 (ATF6)) were analyzed in transcripts per million (TPM). The expression of BIP, CHOP and HERP significantly increased in parallel with the exposure time to RM-581 in THP-1 cells (Figure 9A), as well as the ER transmembrane proteins IRE1α, PERK and ATF6 (Figure 9B). In this study, we did not confirm if the upregulation of these genes was translated into protein expression, but that was already done for CHOP and BIP by immunoblotting in HL-60 leukemia cells treated with the closely related aminosteroid RM-133 [33].

Taken together, these results demonstrate that RM-581 disrupts the transcriptomic profile of THP-1 cells by upregulating and downregulating genes, up to 855 and 415 each, respectively, participating in the ER unfolded protein response and DNA replication.

## 4. Discussion

The aminosteroid RM-581 demonstrated promising anticancer activity against a large spectrum of solid-tumor cancer cell lines, including those with poor prognosis [10,11,12]. However, this new promising molecule had yet to be tested on leukemia, a cancer affecting a vast number of individuals worldwide. In this study, we assessed its anticancer effect on six leukemia cell lines.

RM-581 showed antiproliferative activity (IC_50_ values) in the low micromolar range for multiple leukemia cell lines, characterizing the four leukemia types (AML, ALL, CML and CLL). This suggests that RM-581 may act on a shared vulnerability among leukemias with distinct mutations, such as coordinating bodies (C-bodies) characterized by Riback and Goodell labs [34]. However, the IC_50_ values are in the same range as most solid-cancer cell lines (breast, pancreas, and prostate) already tested by our team, suggesting a mechanism of action more specific to cancer cells [10,12,35].

Combination regimens are a current standard of care for leukemia, such as a combination of the BCL2 inhibitor venetoclax and azacitidine for AML patients or a combination of tyrosine kinase inhibitors and chemotherapy for ALL patients [36,37]. Doxorubicin, an anthracycline used as a chemotherapy drug, can be administered alone or with other drugs to treat ALL, AML and other cancers. It blocks the enzyme topoisomerase II, which stops the process of replication by preventing the DNA double helix from being resealed after the break of the DNA chain [38,39]. Although doxorubicin was more active than RM-581, the combination results show that our compound still contributed to the overall effect. The three myeloid leukemia cell lines (AML: HL-60 and THP-1; CML: K-562) shifted from additivity at low Fa to antagonism at high Fa, which suggests that RM-581 may act through a different pathway than doxorubicin. Also, the ALL cell line (JURKAT) maintained an additive effect across all Fa, indicating that interactions between the two drugs may depend on the cellular context of each leukemia subtype. Overall, these results support that RM-581 may have an independent mechanism from doxorubicin and remain active even when combined with a stronger drug.

Analysis of PBL subpopulations treated with RM-581 revealed a significant decrease in T-lymphocytes, especially in the CD8+ T cytotoxic subpopulation, while the CD4+ T helper subpopulation was not affected. This result differs from the known immune response profile of chemotherapy drugs, which typically induce a sustained depletion of CD4+ T lymphocytes [40,41]. The decrease in T-lymphocytes was observed only at 50 µM, suggesting that RM-581 affects the T-lymphocyte population only at higher concentrations. Considering that the cytotoxic activity of RM-581 is in the low-micromolar range, the fact that T-lymphocytes only decrease at higher doses suggests that this effect is unlikely to be relevant at therapeutic concentrations. Additionally, previous in vivo studies reported no toxicity in mice [16,18], even at doses as high as 720 mg/kg [10], supporting the idea that RM-581 does not broadly impair the immune system.

THP-1 cells were selected for further investigation due to their relevance as an AML model and their widespread use in studies of therapeutic responses [29]. In this cell line, RM-581 (IC_50_ = 5.61 µM) demonstrated higher apparent potency than cytarabine, a standard AML therapeutic, which has been reported to exhibit an IC_50_ of 53.6 µM under comparable conditions [42]. These data support the relevance of this model for subsequent mechanistic investigations.

An important parameter in the evaluation of anticancer agents is their ability to induce one of two forms of cancer cell death: apoptosis or necrosis. In line with previous studies, flow cytometry experiments confirmed the proapoptotic effect of RM-581 at 10 µM, with more than half of THP-1 cells entering apoptosis at 72 h with no involvement of necrosis [10,16]. One way for cancer cells to enter apoptosis is through cell cycle arrest, either at a DNA damage checkpoint (interphase) or the DNA replication stress checkpoint (S-phase). The DNA damage checkpoint can induce irreversible exit from the cell cycle during its multiple phases, including the G1-phase and S-phase [43]. RM-581 induced a G0/G1 cell cycle block, as early as 24 h post-treatment, which lasted for at least 72 h. The proteins regulating the G1 progression and G1/S transition of the cell cycle were analyzed. Cyclins form complexes with CDKs to progress in the cell cycle, while CKIs inhibit the activity of CDKs and cyclin–CDK complexes. The cyclin D–CDK4 or cyclin D–CDK6 complex is necessary to progress through the G1-phase. Meanwhile, cyclin A–CDK2 and cyclin E–CDK2 complexes regulate G1/S transition [44,45]. RM-581 induced a downregulation in the CDK2, cyclin E and cyclin A isoform expressions, as well as in key cell cycle markers, such as Ki-67, PCNA and the MCM protein complex (MCM2-7). This last group is crucial to ensure that DNA replication is only initiated once per cell cycle, during the G1-phase [46]. Taken together, these results suggest that RM-581 suppresses entry into the S-phase. Simultaneously, CKIs p21 and p27 levels, which are known to prevent entry into the S-phase by inhibiting cyclin–CDK complexes [43], were increased, which likely reduced CDK2 and CDK4/6 activity. Additionally, the absence of changes in cyclin D, p16 and p53 expression tends to imply that the arrest is independent of the p53 pathway or p16 pathway, which suggests that our compound acts downstream, or in parallel, to the cyclin D regulation pathway [47]. Altogether, these results reflect the possibility that RM-581 triggers a G1 arrest, leading to a failure to initiate DNA synthesis and preventing the S-phase entry.

Previous studies have identified RM-581 as an ER stress aggravator that can cause ER stress-induced apoptosis [12,16,18]. The ER is a crucial organelle that ensures the quality of production and folding of cellular proteins, contributing to cell homeostasis. However, when the demand for protein increases and exceeds capacity, unfolded proteins can accumulate in the ER lumen and trigger an ER stress. This stress leads to the activation of an adaptive response called unfolded protein response (UPR), whose role is to maintain protein homeostasis [48,49]. An increase in BIP (also known as GRP78) and HERP, two ER stress-related genes, was observed in the RNA-seq analysis of THP-1 cells treated with RM-581. BIP is an essential ER chaperone that maintains normal function of the ER and can trigger the UPR when it dissociates from the UPR sensors [49]. HERP, meanwhile, is an ER membrane protein that contributes to the efficiency of the ER-associated degradation (ERAD) mechanism [50]. Additionally, the level of CHOP, a UPR downstream effector that activates apoptosis induced by ER stress, increases with RM-581 treatment. The UPR is regulated by three ER sensors: IRE1α, PERK and ATF6. The activation of those effectors can lead to two signaling pathways: first, adaptive UPR and, second, proapoptotic UPR [48,49,50,51,52]. Transcriptomic analyses showed the expression of all three effectors is upregulated in THP-1 cells treated with RM-581. This suggests that the UPR is activated as early as 6 h post-treatment, demonstrating that RM-581 rapidly induces stress in cancer cells, which initiates the adaptive UPR that progresses toward the pro-apoptotic response [52]. Taken together, these results concur with previous results to present RM-581 as an ER stress aggravator producing ER stress-induced apoptosis.

To comprehend the mechanism of RM-581, an RNA-seq analysis was conducted. At 24 h, compared with non-treated cells, RM-581 induced an upregulation of up to 855 genes and downregulation of up to 415 genes. This analysis confirms what was observed by flow cytometry and mRNA levels. As both processes are activated at 6 h, the question is whether RM-581 induces ER stress that causes a cell cycle arrest or if it induces the cell cycle arrest that causes ER stress, both of which ultimately lead to apoptosis. Considering that previous experiments using RM-581-Fluo as a fluorescent probe showed accumulation of RM-581 in the ER and none in the nucleus [14,16], the evidence points to ER stress being activated initially, generating stress that may result in the inhibition of DNA replication with a G0/G1 arrest. When ER stress activates the UPR, its sensors, like PERK, inhibit protein synthesis and can halt the cell cycle to reduce protein load to try to reestablish ER homeostasis or to trigger apoptosis if the stress is prolonged [53,54,55]. Further investigations are needed to completely elucidate the mechanism of action of RM-581 and to ultimately identify its molecular therapeutic target.

We have demonstrated the efficacy of RM-581 in vitro across six leukemic cell lines and provided initial insight into its mechanism of action. However, an important limitation of the present study is the absence of in vivo validation in leukemia models. Future studies will, therefore, focus on evaluating RM-581 in relevant leukemia xenograft models to assess its antitumor efficacy, tolerability, and pharmacokinetic/pharmacodynamic relationships. Notably, the related aminosteroid RM-133 has previously demonstrated partial tumor growth inhibition (57% after 39 days) in an HL-60 leukemia xenograft model without observable toxicity [56], supporting the in vivo feasibility of this chemical class. In addition, RM-581 has shown antitumor activity in multiple solid tumor xenograft models and exhibits improved oral bioavailability relative to RM-133 [10,16,17,18]. Together, these findings support the continued preclinical development of RM-581, although additional investigation in vivo leukemia studies will be required to confirm its therapeutic potential in cancer therapy.

## 5. Conclusions

In this study, it was shown that the aminosteroid RM-581 exhibited antiproliferative activity in six leukemia cells resembling that observed in solid tumor cell lines. In human PBLs, RM-581 impacted T lymphocytes, particularly cytotoxic T cells, at 50 µM only, suggesting that this effect might be irrelevant at therapeutic concentrations. In selected THP-1 cells, RM-581 induced apoptosis and G0/G1 cell cycle arrest, with transcriptomic analysis, suggesting a role as an ER stress inducer leading to this response. With that new information, we aim to expand our understanding of the mechanism of action of RM-581 in leukemia and to test its efficacy in leukemia mouse models, while continuing our research on solid tumor cancers, including some with poor prognosis.

## Figures and Tables

**Table 1:** Viability screening experiments with RM-581 in various leukemia cell lines.

Cell Lines	Category	IC_50_ (µM) ^1^ RM-581	IC_50_ (µM) ^1^ Doxorubicin
HL-60	AML	1.71 ± 1.09	0.36 ± 0.27
THP-1	AML	5.61 ± 0.67	0.21 ± 0.13
JURKAT	ALL	1.18 ± 0.51	0.62 ± 0.54
K-562	CML	4.47 ± 0.77	0.88 ± 0.58
HG-3	CLL	2.49 ± 0.64	0.027 ± 0.025
JVM-2	CLL	2.71 ± 0.58	0.017 ± 0.017

^1^ Cell proliferation was assessed after 3 days of treatment using MTS. The values are the means of 3–5 experiments performed in triplicate ± SD.

## Data Availability

The data are contained within this article and the Supplementary Materials.

## Supplementary Materials

The following supporting information can be downloaded at https://www.mdpi.com/article/10.3390/cancers18071078/s1， Figure S1: Effect of doxorubicin on viable, early apoptotic (EAp), late apoptotic (LAp) and necrotic (Ne) THP-1 cells; Figure S2: MCM2-7 gene expression profiles in THP-1 cells exposed for 6 h, 12 h and 24 h to 10 µM of RM-581 analyzed by mRNA sequencing and normalized by transcripts per million (TPM); Figure S3: Heatmap of differentially expressed genes (DEGs) of interest identified from mRNA-seq data; Table S1: (A) Proportion (%) of viable single cells (PBLs) measured by flow cytometry; (B) Proportion (%) of viable THP-1 cells measured by flow cytometry; Table S2: Top 50 of genes upregulated or downregulated at 6 h post-RM-581 treatment in THP-1 cells (padj ≤ 0.05); Table S3: Top 50 of genes upregulated or downregulated at 12 h post-RM-581 treatment in THP-1 cells (padj ≤ 0.05); Table S4: Top 50 of genes upregulated or downregulated at 24 h post-RM-581 treatment in THP-1 cells (padj ≤ 0.05); Table S5: List of STRING-enriched GO biological process enrichment of upregulated genes at 6 h; Table S6: List of STRING-enriched GO biological process enrichment of upregulated genes at 12 h; Table S7: List of STRING-enriched GO biological process enrichment of upregulated genes at 24 h; Table S8: List of STRING-enriched GO biological process enrichment of downregulated genes at 12 h; Table S9: List of STRING-enriched GO biological process enrichment of downregulated genes at 24 h.

## Abbreviations

The following abbreviations were used in this manuscript:

ALL	Acute Lymphoblastic Leukemia
AML	Acute Myeloid Leukemia
ATF6	Activating Transcription Factor 6
ATRA	All-Trans-Retinoic Acid
BPs	Biological Processes
C-bodies	Coordinating Bodies
CDKs	Cyclin-Dependent Kinases
CI	Combination Index
CKIs	Cyclin-Dependent Kinase Inhibitors
CLL	Chronic Lymphocytic Leukemia
CML	Chronic Myeloid Leukemia
DEGs	Differential Expressed Genes
DMSO	Dimethylsulfoxide
ER	Endoplasmic Reticulum
ERAD	ER-Associated Degradation
Fa	Fraction Affected
IARC	International Agency for Research on Cancer
IC50	50% Inhibitory Concentration of Cell Proliferation
IRE1α	Inositol-Requiring Enzyme 1 Alpha
MTS	3-(4,5-dimethylthiazol-2-yl)-5-(3-carboxymethoxyphenyl)-2-(4-sulfophenyl)-2H-tetrazolium
PBLs	Normal Peripheral Blood Lymphocytes
PBMCs	Normal Peripheral Blood Mononuclear Cells
PBS	Phosphate-Buffered Saline
PCA	Principal Component Analysis
PERK	Protein Kinase RNA-Like ER Kinase
PHA-L	Phytohemagglutinin-L
PI	Propidium Iodide
TPM	Transcripts Per Million
UPR	Unfolded Protein Response
