# Supplementary material for: Aminosteroid RM-581 Induces G0/G1 Arrest and Endoplasmic Reticulum Stress-Mediated Apoptosis in Human Acute and Chronic Leukemia Cell Lines
Source: Cancers (Basel). 2026 Mar 26;18(7):1078. doi: 10.3390/cancers18071078 (PMC13072159; doi:10.3390/cancers18071078)
Supplement: Supplementary file 1 [file cancers-18-01078-s001.zip › cancers-4191892-supplementary.pdf]

SUPPLEMENTARY MATERIALS

Aminosteroid RM-581 induces G0/G1 arrest and endoplasmic reticulum stress-mediated apoptosis in acute and chronic leukemia cell lines

Maude Fleury [1,2,3], Jenny Roy [1], René Maltais [1], Francine Durocher [1,2,3] and Donald Poirier [1,2,3,*]

[1]Endocrinology and Nephrology Unit, CHU de Québec Research Center-Université Laval, Pavillon CHUL, Québec, QC G1V 4G2, Canada
[2]Department of Molecular Medicine, Faculty of Medicine, Université Laval, Québec, QC G1V 0A6, Canada
[3] Cancer Research Center, CHU de Québec-Université Laval Research Center, Québec, QC G1R 3S3, Canada

*Correspondence: donald.poirier@crchudequebec.ulaval.ca; Tel.: +1-418-654-2296; Fax: +1-418-654-2298

<u>**Content**</u>:

Figure S1. Effect of doxorubicin on viable, early apoptotic (EAp), late apoptotic (LAp) and necrotic (Ne) THP-1 cells

Figure S2. MCM2-7 gene expression profiles in THP-1 cells

Figure S3. Heatmap of differentially expressed genes (DEGs) of interest identified from mRNA-seq data in THP-1 cells

Table S1A. Proportion (%) of viable single cells (PBLs) measured by flow cytometry

Table S1B. Proportion (%) of viable single cells (THP-1) measured by flow cytometry

Table S2. Top 50 of gene upregulated or downregulated at 6 h post RM-581 treatment in THP-1 cells (padj ≤ 0.05)

Table S3. Top 50 of gene upregulated or downregulated at 12 h post RM-581 treatment in THP-1 cells (padj ≤ 0.05)

Table S4. Top 50 of gene upregulated or downregulated at 24 h post RM-581 treatment in THP-1 cells (padj ≤ 0.05)

Table S5. List of STRING-enriched GO biological process enrichment of upregulated genes at 6 h

Table S6. List of STRING-enriched GO biological process enrichment of upregulated genes at 12 h

Table S7. List of STRING-enriched GO biological process enrichment of upregulated genes at 24 h

Table S8. List of STRING-enriched GO biological process enrichment of downregulated genes at 12 h

Table S9. List of STRING-enriched GO biological process enrichment of downregulated genes at 24 h

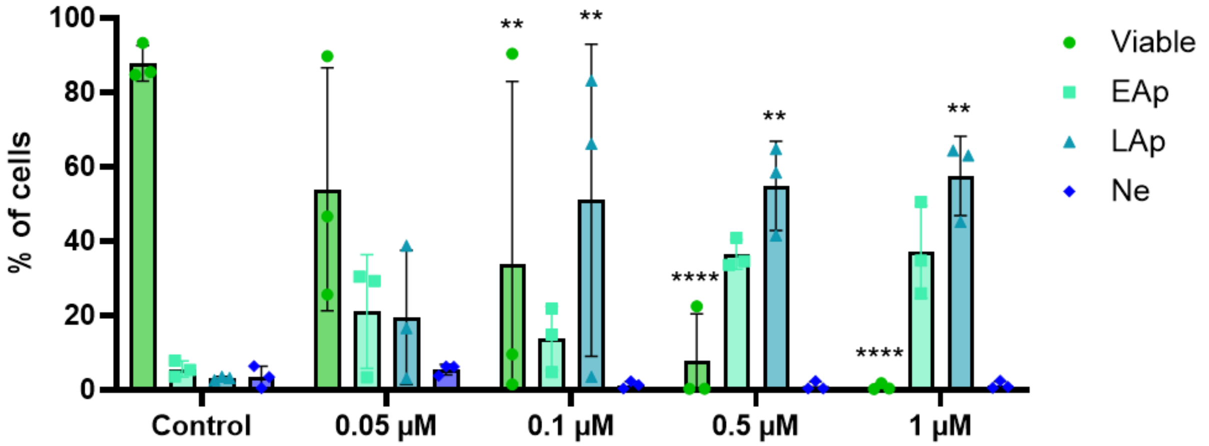

Figure S1. Effect of doxorubicin on viable, early apoptotic (EAp), late apoptotic (LAp) and necrotic (Ne) THP-1 cells. The cells (2 x 10^5) were exposed to doxorubicin for 72 h at different concentrations (0.05, 0.1, 0.5 and 1.0 µM) and flow cytometric analysis using annexin-V and propidium iodide dyes allowed for the determination of each cell type. Each data point represents the mean of three independent experiments (mean ± SD). 2-way ANOVA. * $p < 0.05$, ** $p < 0.005$, *** $p < 0.001$, **** $p < 0.0001$ versus control.

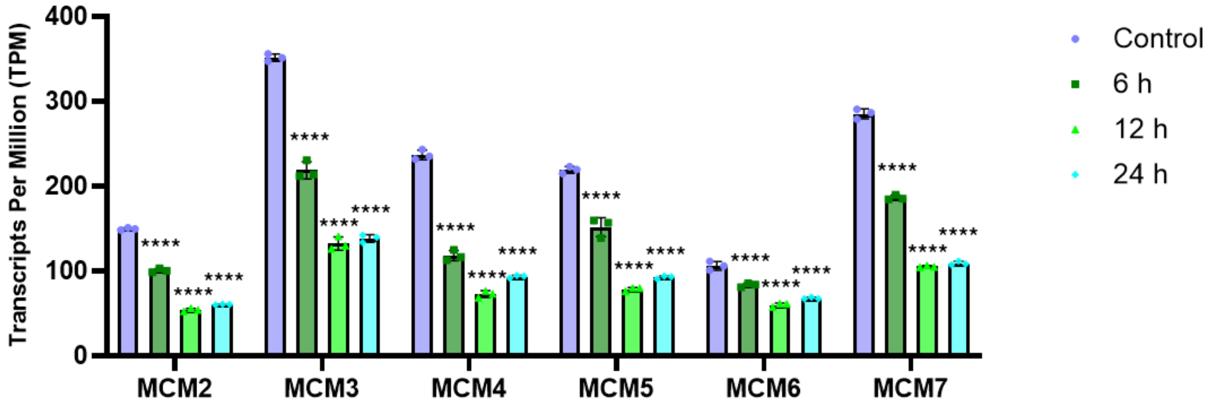

Figure S2. MCM2-7 gene expression profiles in THP-1 cells exposed for 6 h, 12 h and 24 h to 10 µM of RM-581 analyzed by mRNA-sequencing and normalised by transcript per million (TPM). Each data point represents the mean of an experiment performed in triplicate (mean ± SD). 2way ANOVA, Dunnett's multiple comparisons test. * $p < 0.05$, ** $p < 0.005$, *** $p < 0.001$, **** $p < 0.0001$ versus control.

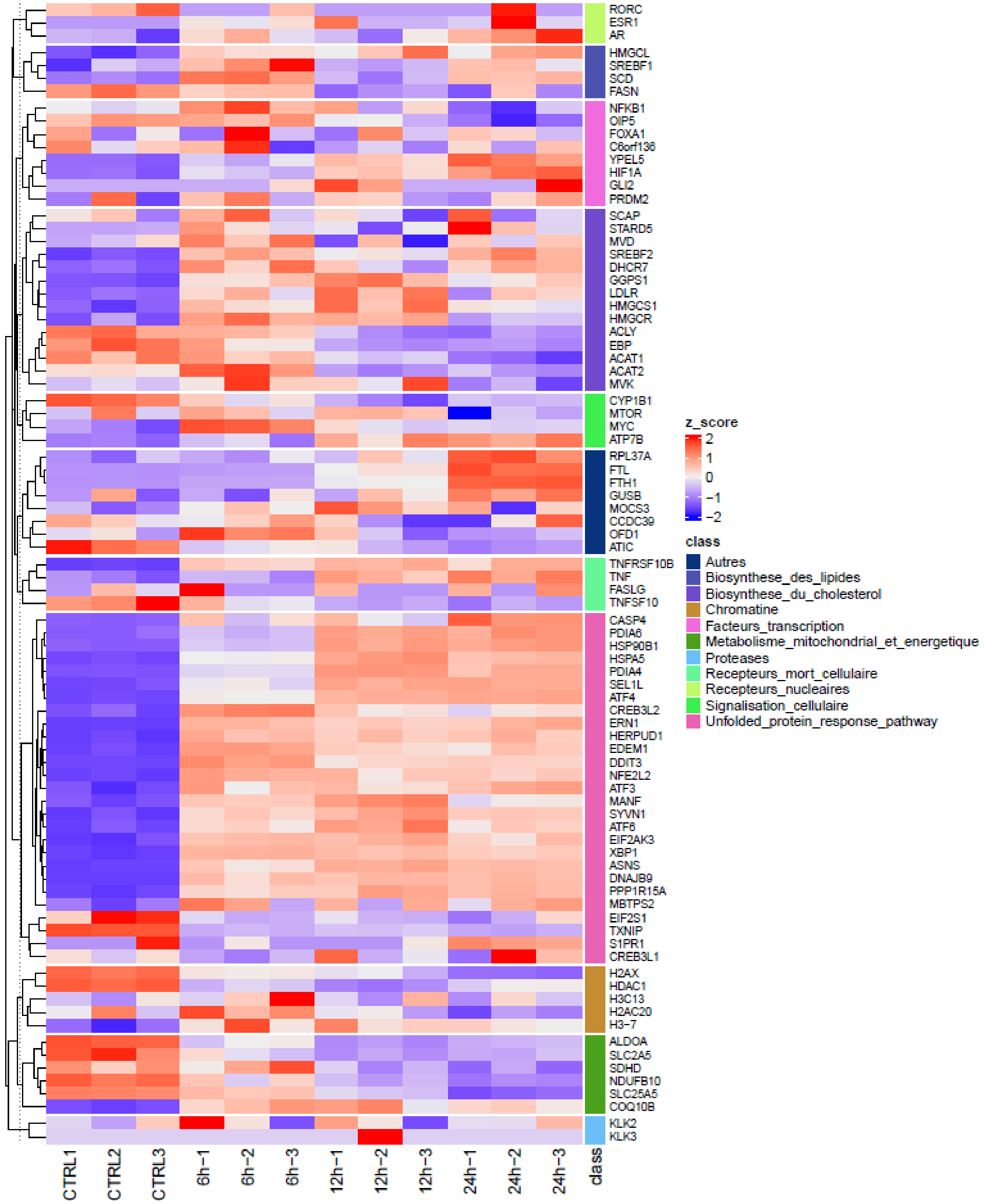

Figure S3. Heatmap of differentially expressed genes (DEGs) of interest identified from mRNA-seq data in THP-1 cells. Expression values were normalized using z-score transformation across each gene (row-wise), where red indicates higher-than-average expression (positive z-score) and blue indicates lower-than-average expression (negative z-score). Each row represents a gene, and each column represents one of the experimental conditions (control, 6 h, 12 h and 24 h).

Table S1A. Proportion (%) of viable single cells (PBLs) measured by flow cytometry [a].

Concentration	Exp. 1	Exp. 2	Exp. 3	Exp. 4
Control	54.9	39.7	40.5	59.3
0.1 µM	48.2	24.5	41.0	43.8
1.0 µM	25.4	24.3	33.9	43.3
5.0 µM	32.4	24.2	29.9	44.2
20 µM	16.6	8.8	18.7	13.2
50 µM	5.4	3.0	3.0	6.3
**IC_{50} (µM)**	**6.9**	**3.9**	**12.6**	**6.3**
**Mean IC_{50} (µM) [b]**		**7.4 ± 3.6**		

[a] Viable cells (1 x 10^5) were determined after 3 days of treatment with RM-581 by the Ghost Dye Red 780, where viable cells exhibited significantly less fluorescence than dead cells, and analyzed by flow cytometry.
[b] The values are the means of 4 experiments (mean ± SD).

Table S1B. Proportion (%) of viable single cells (THP-1) measured by flow cytometry [a].

Concentration	Exp. 1	Exp. 2	Exp. 3
Control	98.8	98.0	92.9
0.1 µM	98.0	91.2	93.5
1.0 µM	97.7	95.8	82.1
5.0 µM	94.7	88.5	60.3
10 µM	92.3	94.8	93.4 [b]
50 µM	1.2	0.04	0.01
**IC_{50} (µM)**	**18.7**	**19.0**	**7.03**
**Mean IC_{50} (µM) [c]**		**14.9 ± 6.8**	

[a] Viable cells (1 x 10^5) were determined after 3 days of treatment with RM-581 by the Ghost Dye Red 780, where viable cells exhibited significantly less fluorescence than dead cells, and analyzed by flow cytometry.
[b] The viability value at 10 µM in Exp. 3 was excluded from IC_{50} calculation due to an aberrant viability measurement, inconsistent with the expected dose-response pattern.
[c] The values are the means of 3 experiments (mean ± SD).

Table S2. Top 50 of gene upregulated or dowregulated at 6 h post RM-581 treatment in THP-1 cells (padj ≤ 0.05).

Upregulated			Downregulated		
ensembl_gene	symbol	log2FoldChange	ensembl_gene	symbol	log2FoldChange
ENSG00000113739	STC2	5.92	ENSG00000277044	OPRL1	-4.77
ENSG00000130487	KLHDC7B	5.84	ENSG00000148408	CACNA1B	-3.83
ENSG00000130513	GDF15	5.83	ENSG00000228875	CSNK2B	-3.56
ENSG00000169676	DRD5	5.54	ENSG00000278787	UBR7	-3.06
ENSG00000272636	DOC2B	4.99	ENSG00000204713	TRIM27	-3.02
ENSG00000101890	GUCY2F	4.63	ENSG00000129467	ADCY4	-2.99
ENSG00000128965	CHAC1	4.62	ENSG00000196826		-2.55
ENSG00000131711	MAP1B	4.47	ENSG00000115884	SDC1	-2.16
ENSG00000139269	INHBE	4.31	ENSG00000115041	KCNIP3	-2.03
ENSG00000196517	SLC6A9	4.25	ENSG00000262446	GBA1	-1.97
ENSG00000128165	ADM2	4.2	ENSG00000084628	NKAIN1	-1.95
ENSG00000168209	DDIT4	4.2	ENSG00000179300	RTL3	-1.93
ENSG00000116761	CTH	3.96	ENSG00000176387	HSD11B2	-1.86
ENSG00000101255	TRIB3	3.9	ENSG00000186810	CXCR3	-1.77
ENSG00000176046	NUPR1	3.81	ENSG00000166897	ELFN2	-1.65
ENSG00000130766	SESN2	3.75	ENSG00000127824	TUBA4A	-1.58
ENSG00000285069	SESN2	3.75	ENSG00000160161	CILP2	-1.57
ENSG00000137502	RAB30	3.64	ENSG00000169684	CHRNA5	-1.52
ENSG00000145777	TSLP	3.63	ENSG00000275600	PIGW	-1.5
ENSG00000112183	RBM24	3.62	ENSG00000135740	SLC9A5	-1.49
ENSG00000184261	KCNK12	3.48	ENSG00000110092	CCND1	-1.48
ENSG00000146674	IGFBP3	3.44	ENSG00000159618	ADGRG5	-1.48
ENSG00000122420	PTGFR	3.43	ENSG00000169403	PTAFR	-1.45
ENSG00000221955	SLC12A8	3.36	ENSG00000180758	GPR157	-1.45

ENSG00000278780	ZNF430	3.36	ENSG00000016391	CHDH	-1.4
ENSG00000112715	VEGFA	3.33	ENSG00000134716	CYP2J2	-1.38
ENSG00000184005	ST6GALNAC3	3.33	ENSG00000105245	NUMBL	-1.35
ENSG00000111981	ULBP1	3.29	ENSG00000223481	TAP2	-1.35
ENSG00000155158	TTC39B	3.26	ENSG00000225967	TAP2	-1.35
ENSG00000144481	TRPM8	3.25	ENSG00000285304		-1.35
ENSG00000070669	ASNS	3.2	ENSG00000156966	B3GNT7	-1.34
ENSG00000142405	NLRP12	3.19	ENSG00000175305	CCNE2	-1.32
ENSG00000129474	AJUBA	3.18	ENSG00000269404	SPIB	-1.32
ENSG00000109819	PPARGC1A	3.16	ENSG00000105173	CCNE1	-1.31
ENSG00000151012	SLC7A11	3.12	ENSG00000197632	SERPINB2	-1.31
ENSG00000138835	RGS3	3.08	ENSG00000265972	TXNIP	-1.31
ENSG00000175197	DDIT3	3.02	ENSG00000123405	NFE2	-1.3
ENSG00000168672	LRATD2	2.94	ENSG00000083454	P2RX5	-1.29
ENSG00000104413	ESRP1	2.92	ENSG00000146904	EPHA1	-1.29
ENSG00000174136	RGMB	2.88	ENSG00000284816	EPHA1	-1.29
ENSG00000079156	OSBPL6	2.86	ENSG00000076248	UNG	-1.28
ENSG00000179813	FAM216B	2.86	ENSG00000189057	FAM111B	-1.27
ENSG00000102683	SGCG	2.85	ENSG00000225060	PPP1R18	-1.25
ENSG00000007314	SCN4A	2.84	ENSG00000229998	PPP1R18	-1.25
ENSG00000071242	RPS6KA2	2.83	ENSG00000068078	FGFR3	-1.24
ENSG00000064300	NGFR	2.82	ENSG00000128594	LRRC4	-1.23
ENSG00000198142	SOWAHC	2.82	ENSG00000237344	PBX2	-1.23
ENSG00000047648	ARHGAP6	2.81	ENSG00000276043	UHRF1	-1.23
ENSG00000115963	RND3	2.8	ENSG00000173546	CSPG4	-1.21
ENSG00000214274	ANG	2.78	ENSG00000277781	MROH6	-1.21

Table S3. Top 50 of gene upregulated or dowregulated at 12 h post RM-581 treatment in THP-1 cells (padj ≤ 0.05).

Upregulated			Downregulated		
ensembl_gene	symbol	log2FoldChange	ensembl_gene	symbol	log2FoldChange
ENSG00000234846	NEU1	9.95	ENSG00000277276	OTUB2	-6.23
ENSG00000124194	GDAP1L1	6.78	ENSG00000278458	KANSL1	-5.05
ENSG00000130487	KLHDC7B	6.76	ENSG00000230907	FKBPL	-4.42
ENSG00000138813	C4orf17	6.74	ENSG00000104974	LILRA1	-3.15
ENSG00000256349		6.63	ENSG00000221887	HMSD	-3.03
ENSG00000130513	GDF15	6.52	ENSG00000166897	ELFN2	-2.98
ENSG00000132837	DMGDH	6.42	ENSG00000213722	DDAH2	-2.97
ENSG00000145692	BHMT	6.24	ENSG00000285625		-2.95
ENSG00000140522	RLBP1	6.12	ENSG00000179300	RTL3	-2.84
ENSG00000113739	STC2	6.07	ENSG00000148408	CACNA1B	-2.82
ENSG00000102683	SGCG	5.81	ENSG00000078098	FAP	-2.58
ENSG00000272636	DOC2B	5.57	ENSG00000157399	ARSL	-2.58
ENSG00000169676	DRD5	5.11	ENSG00000128284	APOL3	-2.54
ENSG00000275674		5.1	ENSG00000159387	IRX6	-2.38
ENSG00000130294	KIF1A	4.94	ENSG00000288199	LSP1	-2.36
ENSG00000131711	MAP1B	4.83	ENSG00000161905	ALOX15	-2.16
ENSG00000107187	LHX3	4.65	ENSG00000176884	GRIN1	-2.11
ENSG00000186150	UBL4B	4.56	ENSG00000197632	SERPINB2	-2.1
ENSG00000128965	CHAC1	4.55	ENSG00000187733	AMY1C	-2.08
ENSG00000182885	ADGRG3	4.52	ENSG00000114646	CSPG5	-2.07
ENSG00000139269	INHBE	4.48	ENSG00000119915	ELOVL3	-2.07
ENSG00000101890	GUCY2F	4.45	ENSG00000228875	CSNK2B	-2.06
ENSG00000173210	ABLIM3	4.38	ENSG00000171388	APLN	-2.04
ENSG00000196517	SLC6A9	4.38	ENSG00000176387	HSD11B2	-2.04

ENSG00000078081	LAMP3	4.37	ENSG00000206355	CCHCR1	-2.03
ENSG00000128165	ADM2	4.25	ENSG00000234114	CCHCR1	-2.03
ENSG00000180535	BHLHA15	4.19	ENSG00000095637	SORBS1	-2
ENSG00000168209	DDIT4	4.17	ENSG00000169508	GPR183	-1.98
ENSG00000112183	RBM24	4.16	ENSG00000016391	CHDH	-1.96
ENSG00000129646	QRICH2	4.15	ENSG00000116985	BMP8B	-1.91
ENSG00000278662	GOLGA6L10	4.14	ENSG00000157470	FAM81A	-1.91
ENSG00000173376	NDNF	4.09	ENSG00000242550	SERPINB10	-1.89
ENSG00000166833	NAV2	4.06	ENSG00000239732	TLR9	-1.88
ENSG00000104413	ESRP1	4.03	ENSG00000189057	FAM111B	-1.87
ENSG00000176046	NUPR1	4.03	ENSG00000123405	NFE2	-1.86
ENSG00000101255	TRIB3	4.02	ENSG00000117594	HSD11B1	-1.85
ENSG00000124749	COL21A1	4.01	ENSG00000127824	TUBA4A	-1.85
ENSG00000144481	TRPM8	4	ENSG00000151615	POU4F2	-1.85
ENSG00000204334	ERICH2	3.95	ENSG00000265763	ZNF488	-1.84
ENSG00000007314	SCN4A	3.94	ENSG00000184709	LRRC26	-1.83
ENSG00000149256	TENM4	3.91	ENSG00000133055	MYBPH	-1.82
ENSG00000116183	PAPPA2	3.88	ENSG00000084110	HAL	-1.8
ENSG00000012779	ALOX5	3.87	ENSG00000104738	MCM4	-1.79
ENSG00000184005	ST6GALNAC3	3.87	ENSG00000186810	CXCR3	-1.78
ENSG00000281935	DNAH10	3.82	ENSG00000156966	B3GNT7	-1.77
ENSG00000145777	TSLP	3.77	ENSG00000264230	ANXA8L1	-1.77
ENSG00000146674	IGFBP3	3.72	ENSG00000166086	JAM3	-1.75
ENSG00000112715	VEGFA	3.7	ENSG00000165092	ALDH1A1	-1.74
ENSG00000275736	OSCAR	3.64	ENSG00000196415	PRTN3	-1.72
ENSG00000155158	TTC39B	3.63	ENSG00000115884	SDC1	-1.7

Table S4. Top 50 of gene upregulated or dowregulated at 24 h post RM-581 treatment in THP-1 cells (padj ≤ 0.05).

Upregulated			Downregulated		
ensembl_gene	symbol	log2FoldChange	ensembl_gene	symbol	log2FoldChange
ENSG00000132837	DMGDH	7.44	ENSG00000277276	OTUB2	-5.86
ENSG00000138813	C4orf17	7.28	ENSG00000282735	GARRE1	-5.33
ENSG00000256349		7.28	ENSG00000161939	RNASEK-C17orf49	-5.3
ENSG00000130513	GDF15	7.06	ENSG00000140459	CYP11A1	-5.15
ENSG00000130487	KLHDC7B	6.99	ENSG00000288362	CYP11A1	-5.15
ENSG00000140522	RLBP1	6.91	ENSG00000256646		-5.01
ENSG00000102683	SGCG	6.65	ENSG00000234258	HSPA1L	-4.99
ENSG00000124194	GDAP1L1	6.6	ENSG00000288637		-4.84
ENSG00000113739	STC2	6.01	ENSG00000103034	NDRG4	-4.51
ENSG00000091138	SLC26A3	5.96	ENSG00000078098	FAP	-4.39
ENSG00000070808	CAMK2A	5.7	ENSG00000148408	CACNA1B	-4.28
ENSG00000173210	ABLIM3	5.68	ENSG00000146857	STRA8	-3.83
ENSG00000145692	BHMT	5.67	ENSG00000221887	HMSD	-3.8
ENSG00000272636	DOC2B	5.61	ENSG00000126460	PRRG2	-3.6
ENSG00000169676	DRD5	5.43	ENSG00000157399	ARSL	-3.51
ENSG00000244414	CFHR1	5.41	ENSG00000277044	OPRL1	-3.28
ENSG00000275674		5.29	ENSG00000264230	ANXA8L1	-3.25
ENSG00000172985	SH3RF3	5.28	ENSG00000288199	LSP1	-3.17
ENSG00000186150	UBL4B	5.26	ENSG00000259132		-3.11
ENSG00000163285	GABRG1	5.22	ENSG00000144857	BOC	-3.08
ENSG00000251537		5.17	ENSG00000281988	GRK1	-2.97
ENSG00000243978	RTL9	5.17	ENSG00000165092	ALDH1A1	-2.96
ENSG00000278154	LAIR1	5.14	ENSG00000143546	S100A8	-2.75
ENSG00000112183	RBM24	5.08	ENSG00000187733	AMY1C	-2.69

ENSG00000078081	LAMP3	5	ENSG00000161905	ALOX15	-2.68
ENSG00000278545	MARCHF8	4.99	ENSG00000164821	DEFA4	-2.57
ENSG00000273707	CDKN1C	4.99	ENSG00000285318	DEFA4	-2.57
ENSG00000135773	CAPN9	4.95	ENSG00000196415	PRTN3	-2.55
ENSG00000182885	ADGRG3	4.93	ENSG00000141977	CIB3	-2.54
ENSG00000124391	IL17C	4.87	ENSG00000163220	S100A9	-2.54
ENSG00000130948	HSD17B3	4.83	ENSG00000197632	SERPINB2	-2.53
ENSG00000180509	KCNE1	4.8	ENSG00000157554	ERG	-2.52
ENSG00000178662	CSRNP3	4.8	ENSG00000099956	SMARCB1	-2.51
ENSG00000129646	QRICH2	4.66	ENSG00000169508	GPR183	-2.48
ENSG00000128965	CHAC1	4.64	ENSG00000077274	CAPN6	-2.44
ENSG00000107187	LHX3	4.63	ENSG00000186810	CXCR3	-2.43
ENSG00000139269	INHBE	4.55	ENSG00000082482	KCNK2	-2.39
ENSG00000113722	CDX1	4.54	ENSG00000107796	ACTA2	-2.39
ENSG00000007314	SCN4A	4.52	ENSG00000134873	CLDN10	-2.39
ENSG00000168209	DDIT4	4.52	ENSG00000117594	HSD11B1	-2.38
ENSG00000131711	MAP1B	4.48	ENSG00000183873	SCN5A	-2.36
ENSG00000163993	S100P	4.47	ENSG00000274290	H2BC6	-2.33
ENSG00000196517	SLC6A9	4.47	ENSG00000146216	TTBK1	-2.29
ENSG00000104413	ESRP1	4.41	ENSG00000103196	CRISPLD2	-2.23
ENSG00000101890	GUCY2F	4.4	ENSG00000118997	DNAH7	-2.22
ENSG00000110002	VWA5A	4.4	ENSG00000138131	LOXL4	-2.18
ENSG00000144481	TRPM8	4.39	ENSG00000084110	HAL	-2.15
ENSG00000275736	OSCAR	4.39	ENSG00000242550	SERPINB10	-2.15
ENSG00000128165	ADM2	4.36	ENSG00000267561		-2.15
ENSG00000249662	LINC02218	4.34	ENSG00000119915	ELOVL3	-2.14

Table S5. List of STRING-enriched GO biological process enrichment of upregulated genes at 6 h.

GO-term	Description	Observed gene count	Background gene count	Strength	Signal	False discovery rate
GO:0034976	Response to endoplasmic reticulum stress	25	223	0.73	1.11	8.60e-07
GO:0006986	Response to unfolded protein	17	122	0.83	0.96	2.89e-05
GO:0070059	Intrinsic apoptotic signaling pathway in response to endoplasmic reticulum stress	9	34	1.1	0.78	0.00067
GO:0030968	Endoplasmic reticulum unfolded protein response	10	54	0.95	0.67	0.0017
GO:1905897	Regulation of response to endoplasmic reticulum stress	12	85	0.83	0.64	0.0017
GO:0034620	Cellular response to unfolded protein	11	79	0.83	0.59	0.0031
GO:1900101	Regulation of endoplasmic reticulum unfolded protein response	7	31	1.04	0.5	0.0106
GO:1990440	Positive regulation of transcription from RNA polymerase II promoter in response to endoplasmic reticulum stress	5	12	1.3	0.49	0.0136
GO:0035556	Intracellular signal transduction	62	1518	0.29	0.48	0.0014
GO:0089718	Amino acid import across plasma membrane	7	33	1.01	0.47	0.0136
GO:0015804	Neutral amino acid transport	8	50	0.89	0.46	0.0136
GO:0036003	Positive regulation of transcription from RNA polymerase II promoter in response to stress	6	25	1.06	0.46	0.0165
GO:0042981	Regulation of apoptotic process	59	1462	0.29	0.45	0.0023
GO:0043067	Regulation of programmed cell death	60	1492	0.29	0.45	0.0023
GO:0010941	Regulation of cell death	64	1651	0.27	0.44	0.0026
GO:0097193	Intrinsic apoptotic signaling pathway	14	166	0.61	0.43	0.0136
GO:1901607	Alpha-amino acid biosynthetic process	9	70	0.79	0.43	0.0165
GO:0042149	Cellular response to glucose starvation	8	55	0.84	0.43	0.0190
GO:0042594	Response to starvation	16	206	0.57	0.42	0.0136
GO:0015807	L-amino acid transport	9	73	0.77	0.42	0.0197
GO:1902475	L-alpha-amino acid transmembrane transport	9	73	0.77	0.42	0.0197
GO:0140467	Integrated stress response signaling	6	31	0.97	0.4	0.0274
GO:0043065	Positive regulation of apoptotic process	27	507	0.41	0.39	0.0136
GO:0006865	Amino acid transport	11	116	0.66	0.39	0.0216
GO:2000271	Positive regulation of fibroblast apoptotic process	4	10	1.28	0.38	0.0387
GO:0010942	Positive regulation of cell death	29	590	0.37	0.37	0.0162
GO:0009070	Serine family amino acid biosynthetic process	5	21	1.06	0.37	0.0395
GO:0010033	Response to organic substance	89	2692	0.2	0.36	0.0085
GO:0071496	Cellular response to external stimulus	19	317	0.46	0.36	0.0228
GO:0021772	Olfactory bulb development	6	34	0.93	0.36	0.0387

GO:1902235	Regulation of endoplasmic reticulum stress-induced intrinsic apoptotic signaling pathway	6	34	0.93	0.36	0.0387
GO:0051716	Cellular response to stimulus	176	6357	0.12	0.35	0.0066
GO:0048519	Negative regulation of biological process	152	5313	0.14	0.35	0.0077
GO:0033554	Cellular response to stress	58	1572	0.25	0.35	0.0136
GO:0009991	Response to extracellular stimulus	25	492	0.39	0.35	0.0228
GO:0031667	Response to nutrient levels	24	461	0.4	0.35	0.0228
GO:0031668	Cellular response to extracellular stimulus	16	248	0.49	0.34	0.0314
GO:0006950	Response to stress	104	3358	0.17	0.33	0.0136
GO:0010646	Regulation of cell communication	104	3355	0.17	0.33	0.0136
GO:0023051	Regulation of signaling	104	3367	0.17	0.33	0.0136
GO:0001932	Regulation of protein phosphorylation	44	1108	0.28	0.33	0.0202
GO:0042325	Regulation of phosphorylation	48	1251	0.27	0.33	0.0202
GO:0031399	Regulation of protein modification process	56	1560	0.24	0.32	0.0228
GO:0045598	Regulation of fat cell differentiation	11	135	0.59	0.32	0.0475
GO:0048513	Animal organ development	99	3246	0.17	0.31	0.0203
GO:0070887	Cellular response to chemical stimulus	83	2609	0.18	0.31	0.0228
GO:0010647	Positive regulation of cell communication	59	1693	0.22	0.31	0.0267
GO:0050789	Regulation of biological process	284	11655	0.07	0.3	0.0136
GO:0050794	Regulation of cellular process	271	11025	0.07	0.3	0.0136
GO:0048518	Positive regulation of biological process	168	6207	0.11	0.3	0.0190
GO:0048523	Negative regulation of cellular process	134	4736	0.13	0.3	0.0209
GO:0023056	Positive regulation of signaling	59	1698	0.22	0.3	0.0274
GO:0006520	Cellular amino acid metabolic process	17	290	0.45	0.3	0.0475
GO:0050896	Response to stimulus	203	7835	0.1	0.29	0.0202
GO:0031323	Regulation of cellular metabolic process	155	5681	0.12	0.29	0.0228
GO:0070848	Response to growth factor	24	503	0.36	0.29	0.0494
GO:0065007	Biological regulation	296	12385	0.06	0.28	0.0202
GO:0019222	Regulation of metabolic process	179	6784	0.1	0.28	0.0248
GO:0060255	Regulation of macromolecule metabolic process	167	6249	0.11	0.28	0.0267
GO:0048878	Chemical homeostasis	36	904	0.28	0.28	0.0494
GO:0007154	Cell communication	142	5165	0.12	0.27	0.0329
GO:0048583	Regulation of response to stimulus	113	3931	0.14	0.27	0.0388
GO:0019220	Regulation of phosphate metabolic process	50	1405	0.23	0.27	0.0474
GO:0009967	Positive regulation of signal transduction	53	1525	0.22	0.27	0.0494
GO:0048522	Positive regulation of cellular process	151	5584	0.11	0.26	0.0387
GO:0009966	Regulation of signal transduction	90	2978	0.16	0.26	0.0434
GO:0080090	Regulation of primary metabolic process	157	5899	0.11	0.25	0.0480

Table S6. List of STRING-enriched GO biological process enrichment of upregulated genes at 12 h.

GO-term	Description	Observed gene count	Background gene count	Strength	Signal	False discovery rate
GO:0034620	Cellular response to unfolded protein	18	79	0.84	1.02	1.43e-05
GO:0006986	Response to unfolded protein	22	122	0.74	0.97	1.43e-05
GO:0030968	Endoplasmic reticulum unfolded protein response	14	54	0.9	0.96	4.84e-05
GO:0034976	Response to endoplasmic reticulum stress	29	223	0.6	0.88	1.43e-05
GO:1990440	Positive regulation of transcription from RNA polymerase II promoter in response to endoplasmic reticulum stress	7	12	1.25	0.75	0.0011
GO:0036003	Positive regulation of transcription from RNA polymerase II promoter in response to stress	9	25	1.04	0.73	0.00099
GO:1905897	Regulation of response to endoplasmic reticulum stress	15	85	0.73	0.68	0.00079
GO:0008652	Cellular amino acid biosynthetic process	14	77	0.75	0.66	0.0011
GO:1901607	Alpha-amino acid biosynthetic process	13	70	0.76	0.62	0.0018
GO:0010033	Response to organic substance	145	2692	0.22	0.6	1.43e-05
GO:0089718	Amino acid import across plasma membrane	9	33	0.92	0.6	0.0034
GO:0070887	Cellular response to chemical stimulus	139	2609	0.21	0.59	1.97e-05
GO:0070059	Intrinsic apoptotic signaling pathway in response to endoplasmic reticulum stress	9	34	0.91	0.59	0.0036
GO:1903573	Negative regulation of response to endoplasmic reticulum stress	10	45	0.83	0.58	0.0036
GO:0043618	Regulation of transcription from RNA polymerase II promoter in response to stress	10	49	0.8	0.53	0.0059
GO:0009719	Response to endogenous stimulus	81	1363	0.26	0.52	0.00051
GO:0071310	Cellular response to organic substance	108	2019	0.22	0.49	0.00061
GO:0098657	Import into cell	20	189	0.51	0.49	0.0047
GO:0070848	Response to growth factor	38	503	0.37	0.48	0.0029
GO:0006520	Cellular amino acid metabolic process	26	290	0.44	0.48	0.0040
GO:0006865	Amino acid transport	15	116	0.6	0.48	0.0065
GO:0070972	Protein localization to endoplasmic reticulum	11	66	0.71	0.47	0.0100
GO:0043067	Regulation of programmed cell death	84	1492	0.24	0.46	0.0014
GO:0003013	Circulatory system process	37	493	0.36	0.46	0.0036
GO:0071363	Cellular response to growth factor stimulus	36	473	0.37	0.46	0.0036
GO:0042149	Cellular response to glucose starvation	10	55	0.75	0.46	0.0117
GO:1902235	Regulation of endoplasmic reticulum stress-induced intrinsic apoptotic signaling pathway	8	34	0.86	0.46	0.0132
GO:0010941	Regulation of cell death	90	1651	0.22	0.45	0.0018
GO:0042981	Regulation of apoptotic process	82	1462	0.24	0.45	0.0018

GO:0071495	Cellular response to endogenous stimulus	66	1103	0.26	0.45	0.0026
GO:1901698	Response to nitrogen compound	64	1058	0.27	0.45	0.0026
GO:0042221	Response to chemical	183	4010	0.15	0.44	0.0010
GO:0010243	Response to organonitrogen compound	59	963	0.27	0.44	0.0035
GO:0043523	Regulation of neuron apoptotic process	21	216	0.47	0.44	0.0080
GO:0001932	Regulation of protein phosphorylation	65	1108	0.26	0.43	0.0036
GO:0060548	Negative regulation of cell death	61	1016	0.27	0.43	0.0036
GO:0097501	Stress response to metal ion	6	18	1.01	0.43	0.0196
GO:0007167	Enzyme-linked receptor protein signaling pathway	43	641	0.31	0.42	0.0061
GO:0007169	Transmembrane receptor protein tyrosine kinase signaling pathway	32	425	0.36	0.41	0.0087
GO:0015807	L-amino acid transport	11	73	0.66	0.41	0.0170
GO:1902475	L-alpha-amino acid transmembrane transport	11	73	0.66	0.41	0.0170
GO:0007166	Cell surface receptor signaling pathway	103	2040	0.19	0.4	0.0039
GO:0003018	Vascular process in circulatory system	23	262	0.43	0.4	0.0128
GO:0015804	Neutral amino acid transport	9	50	0.74	0.4	0.0211
GO:0006563	L-serine metabolic process	5	12	1.11	0.4	0.0293
GO:0023051	Regulation of signaling	155	3367	0.15	0.39	0.0034
GO:0006950	Response to stress	154	3358	0.15	0.39	0.0036
GO:0006564	L-serine biosynthetic process	4	6	1.31	0.39	0.0334
GO:0015820	Leucine transport	4	6	1.31	0.39	0.0334
GO:0051493	Regulation of cytoskeleton organization	37	541	0.32	0.38	0.0128
GO:0098739	Import across plasma membrane	16	152	0.51	0.38	0.0207
GO:0010043	Response to zinc ion	9	52	0.73	0.38	0.0264
GO:0009070	Serine family amino acid biosynthetic process	6	21	0.94	0.38	0.0322
GO:1902236	Negative regulation of endoplasmic reticulum stress-induced intrinsic apoptotic signaling pathway	6	21	0.94	0.38	0.0322
GO:0051716	Cellular response to stimulus	262	6357	0.1	0.37	0.0033
GO:0050896	Response to stimulus	311	7835	0.09	0.37	0.0036
GO:0062197	Cellular response to chemical stress	23	272	0.41	0.37	0.0172
GO:0140467	Integrated stress response signaling	7	31	0.84	0.37	0.0322
GO:0010273	Detoxification of copper ion	5	14	1.04	0.37	0.0379
GO:0010646	Regulation of cell communication	152	3355	0.14	0.36	0.0061
GO:0043066	Negative regulation of apoptotic process	53	891	0.26	0.36	0.0121
GO:0022603	Regulation of anatomical structure morphogenesis	54	920	0.26	0.36	0.0132
GO:0042594	Response to starvation	19	206	0.45	0.36	0.0234
GO:0071294	Cellular response to zinc ion	6	23	0.9	0.36	0.0379
GO:0048519	Negative regulation of biological process	222	5313	0.11	0.35	0.0069
GO:0042325	Regulation of phosphorylation	68	1251	0.22	0.35	0.0132
GO:0048878	Chemical homeostasis	53	904	0.26	0.35	0.0151

GO:0042592	Homeostatic process	74	1406	0.21	0.34	0.0151
GO:0051240	Positive regulation of multicellular organismal process	78	1505	0.2	0.34	0.0151
GO:0003333	Amino acid transmembrane transport	12	98	0.57	0.34	0.0334
GO:0051897	Positive regulation of protein kinase B signaling	13	113	0.55	0.34	0.0334
GO:0044344	Cellular response to fibroblast growth factor stimulus	11	84	0.6	0.34	0.0350
GO:0008543	Fibroblast growth factor receptor signaling pathway	9	58	0.68	0.34	0.0379
GO:0150104	Transport across blood-brain barrier	11	86	0.59	0.34	0.0379
GO:1901214	Regulation of neuron death	25	324	0.37	0.33	0.0284
GO:0032956	Regulation of actin cytoskeleton organization	27	365	0.36	0.33	0.0293
GO:0048522	Positive regulation of cellular process	229	5584	0.1	0.32	0.0132
GO:0033554	Cellular response to stress	80	1572	0.19	0.32	0.0196
GO:1901700	Response to oxygen-containing compound	79	1547	0.2	0.32	0.0196
GO:0051896	Regulation of protein kinase B signaling	16	166	0.47	0.32	0.0370
GO:1901605	Alpha-amino acid metabolic process	18	202	0.44	0.32	0.0375
GO:0048518	Positive regulation of biological process	250	6207	0.09	0.31	0.0151
GO:1901652	Response to peptide	30	437	0.32	0.31	0.0350
GO:0036294	Cellular response to decreased oxygen levels	14	136	0.5	0.31	0.0433
GO:0035556	Intracellular signal transduction	77	1518	0.19	0.3	0.0264
GO:0001568	Blood vessel development	33	505	0.3	0.3	0.0376
GO:0009966	Regulation of signal transduction	133	2978	0.14	0.29	0.0264
GO:0019220	Regulation of phosphate metabolic process	72	1405	0.2	0.29	0.0311
GO:0031399	Regulation of protein modification process	78	1560	0.19	0.29	0.0322
GO:0010647	Positive regulation of cell communication	83	1693	0.18	0.29	0.0334
GO:0010628	Positive regulation of gene expression	61	1146	0.21	0.29	0.0338
GO:0006082	Organic acid metabolic process	49	868	0.24	0.29	0.0379
GO:0032970	Regulation of actin filament-based process	28	406	0.33	0.29	0.0447
GO:0001525	Angiogenesis	24	325	0.36	0.29	0.0456
GO:0009636	Response to toxic substance	19	229	0.41	0.29	0.0488
GO:0007154	Cell communication	211	5165	0.1	0.28	0.0293
GO:0048583	Regulation of response to stimulus	167	3931	0.12	0.28	0.0311
GO:0051128	Regulation of cellular component organization	109	2365	0.15	0.28	0.0334
GO:0023056	Positive regulation of signaling	83	1698	0.18	0.28	0.0350
GO:0009628	Response to abiotic stimulus	59	1107	0.21	0.28	0.0379
GO:0007275	Multicellular organism development	176	4209	0.11	0.27	0.0356
GO:0009605	Response to external stimulus	108	2355	0.15	0.27	0.0375
GO:0009888	Tissue development	83	1723	0.17	0.27	0.0456
GO:0051239	Regulation of multicellular organismal process	122	2749	0.13	0.26	0.0447
GO:0065008	Regulation of biological quality	155	3654	0.11	0.26	0.0447
GO:0048513	Animal organ development	140	3246	0.12	0.26	0.0468
GO:0009967	Positive regulation of signal transduction	75	1525	0.18	0.26	0.0488

Table S7. List of STRING-enriched GO biological process enrichment of upregulated genes at 24 h.

GO-term	Description	Observed gene count	Background gene count	Strength	Signal	False discovery rate
GO:0034976	Response to endoplasmic reticulum stress	33	223	0.57	0.93	3.57e-06
GO:0030968	Endoplasmic reticulum unfolded protein response	14	54	0.81	0.83	0.00016
GO:0006986	Response to unfolded protein	21	122	0.63	0.79	9.55e-05
GO:0034620	Cellular response to unfolded protein	16	79	0.7	0.74	0.00033
GO:0035966	Response to topologically incorrect protein	22	144	0.58	0.71	0.00025
GO:1990440	Positive regulation of transcription from RNA polymerase II promoter in response to endoplasmic reticulum stress	7	12	1.16	0.71	0.0015
GO:0043067	Regulation of programmed cell death	112	1492	0.27	0.69	3.16e-06
GO:0010941	Regulation of cell death	121	1651	0.26	0.68	3.16e-06
GO:0036003	Positive regulation of transcription from RNA polymerase II promoter in response to stress	9	25	0.95	0.68	0.0015
GO:0042981	Regulation of apoptotic process	108	1462	0.26	0.67	4.81e-06
GO:0060548	Negative regulation of cell death	81	1016	0.3	0.66	1.84e-05
GO:0010033	Response to organic substance	175	2692	0.21	0.64	3.16e-06
GO:0070887	Cellular response to chemical stimulus	170	2609	0.21	0.64	3.16e-06
GO:0035967	Cellular response to topologically incorrect protein	17	99	0.63	0.64	0.00095
GO:0009966	Regulation of signal transduction	186	2978	0.19	0.62	3.16e-06
GO:0009967	Positive regulation of signal transduction	108	1525	0.25	0.62	2.39e-05
GO:0023051	Regulation of signaling	203	3367	0.18	0.6	4.81e-06
GO:0010647	Positive regulation of cell communication	116	1693	0.23	0.6	2.85e-05
GO:0023056	Positive regulation of signaling	116	1698	0.23	0.6	3.13e-05
GO:0048583	Regulation of response to stimulus	229	3931	0.16	0.59	4.81e-06
GO:0010646	Regulation of cell communication	201	3355	0.17	0.59	6.87e-06
GO:0071310	Cellular response to organic substance	133	2019	0.21	0.59	2.45e-05
GO:0006950	Response to stress	200	3358	0.17	0.58	1.00e-05
GO:0043066	Negative regulation of apoptotic process	70	891	0.29	0.58	0.00016
GO:0043069	Negative regulation of programmed cell death	71	911	0.29	0.58	0.00016
GO:1905897	Regulation of response to endoplasmic reticulum stress	15	85	0.64	0.58	0.0023
GO:1902531	Regulation of intracellular signal transduction	116	1726	0.22	0.57	6.45e-05
GO:0051240	Positive regulation of multicellular organismal process	104	1505	0.24	0.57	8.65e-05
GO:0062197	Cellular response to chemical stress	30	272	0.44	0.57	0.00096
GO:0048519	Negative regulation of biological process	289	5313	0.13	0.56	8.81e-06
GO:0043618	Regulation of transcription from RNA polymerase II promoter in response to stress	11	49	0.75	0.54	0.0045
GO:0009719	Response to endogenous stimulus	94	1363	0.23	0.53	0.00030

GO:1902533	Positive regulation of intracellular signal transduction	74	997	0.27	0.53	0.00041
GO:0051716	Cellular response to stimulus	331	6357	0.11	0.52	2.45e-05
GO:0048518	Positive regulation of biological process	324	6207	0.11	0.52	2.74e-05
GO:0043523	Regulation of neuron apoptotic process	25	216	0.46	0.52	0.0026
GO:0042221	Response to chemical	224	4010	0.14	0.51	8.46e-05
GO:0070059	Intrinsic apoptotic signaling pathway in response to endoplasmic reticulum stress	9	34	0.82	0.51	0.0075
GO:0050896	Response to stimulus	391	7835	0.09	0.5	4.13e-05
GO:0048523	Negative regulation of cellular process	256	4736	0.13	0.5	9.55e-05
GO:0048584	Positive regulation of response to stimulus	132	2131	0.19	0.5	0.00036
GO:0015807	L-amino acid transport	13	73	0.65	0.5	0.0063
GO:1902475	L-alpha-amino acid transmembrane transport	13	73	0.65	0.5	0.0063
GO:0051239	Regulation of multicellular organismal process	161	2749	0.16	0.48	0.00043
GO:0010628	Positive regulation of gene expression	80	1146	0.24	0.48	0.0011
GO:0048522	Positive regulation of cellular process	290	5584	0.11	0.47	0.00025
GO:0051128	Regulation of cellular component organization	142	2365	0.17	0.47	0.00060
GO:0070848	Response to growth factor	43	503	0.33	0.47	0.0027
GO:0071363	Cellular response to growth factor stimulus	41	473	0.33	0.47	0.0031
GO:0002931	Response to ischemia	11	55	0.7	0.47	0.0090
GO:2001233	Regulation of apoptotic signaling pathway	34	365	0.36	0.46	0.0042
GO:0036503	ERAD pathway	15	100	0.57	0.46	0.0083
GO:0007154	Cell communication	270	5165	0.11	0.45	0.00043
GO:0051897	Positive regulation of protein kinase B signaling	16	113	0.55	0.45	0.0085
GO:0035556	Intracellular signal transduction	98	1518	0.21	0.44	0.0018
GO:0071495	Cellular response to endogenous stimulus	76	1103	0.23	0.44	0.0026
GO:0022603	Regulation of anatomical structure morphogenesis	66	920	0.25	0.44	0.0030
GO:0043068	Positive regulation of programmed cell death	43	519	0.31	0.44	0.0047
GO:1901214	Regulation of neuron death	31	324	0.38	0.44	0.0058
GO:0051896	Regulation of protein kinase B signaling	20	166	0.48	0.44	0.0083
GO:0006865	Amino acid transport	16	116	0.54	0.44	0.0100
GO:1901607	Alpha-amino acid biosynthetic process	12	70	0.63	0.44	0.0122
GO:0065008	Regulation of biological quality	200	3654	0.13	0.43	0.0011
GO:0001932	Regulation of protein phosphorylation	76	1108	0.23	0.43	0.0029
GO:0007155	Cell adhesion	68	965	0.24	0.43	0.0037
GO:0010942	Positive regulation of cell death	47	590	0.3	0.43	0.0049
GO:0030433	Ubiquitin-dependent ERAD pathway	13	82	0.6	0.43	0.0124
GO:0015804	Neutral amino acid transport	10	50	0.7	0.43	0.0143
GO:0009611	Response to wounding	38	444	0.33	0.42	0.0068
GO:0023052	Signaling	261	5057	0.11	0.41	0.0016
GO:0050793	Regulation of developmental process	144	2492	0.16	0.41	0.0027

GO:0045598	Regulation of fat cell differentiation	17	135	0.5	0.41	0.0136
GO:0003333	Amino acid transmembrane transport	14	98	0.55	0.41	0.0157
GO:0089718	Amino acid import across plasma membrane	8	33	0.78	0.41	0.0213
GO:0032970	Regulation of actin filament-based process	35	406	0.33	0.4	0.0100
GO:0015849	Organic acid transport	27	278	0.38	0.4	0.0112
GO:0036294	Cellular response to decreased oxygen levels	17	136	0.49	0.4	0.0143
GO:0051493	Regulation of cytoskeleton organization	43	541	0.3	0.39	0.0092
GO:0043065	Positive regulation of apoptotic process	41	507	0.3	0.39	0.0094
GO:0003013	Circulatory system process	40	493	0.3	0.39	0.0102
GO:1905039	Carboxylic acid transmembrane transport	21	193	0.43	0.39	0.0143
GO:1902235	Regulation of endoplasmic reticulum stress-induced intrinsic apoptotic signaling pathway	8	34	0.77	0.39	0.0245
GO:0097501	Stress response to metal ion	6	18	0.92	0.39	0.0290
GO:0007165	Signal transduction	243	4714	0.11	0.38	0.0037
GO:0042325	Regulation of phosphorylation	81	1251	0.21	0.38	0.0076
GO:0032956	Regulation of actin cytoskeleton organization	32	365	0.34	0.38	0.0130
GO:1902903	Regulation of supramolecular fiber organization	33	382	0.33	0.38	0.0130
GO:0042149	Cellular response to glucose starvation	10	55	0.66	0.38	0.0248
GO:1903573	Negative regulation of response to endoplasmic reticulum stress	9	45	0.7	0.38	0.0264
GO:0007166	Cell surface receptor signaling pathway	120	2040	0.17	0.37	0.0064
GO:0033554	Cellular response to stress	97	1572	0.19	0.37	0.0075
GO:0045071	Negative regulation of viral genome replication	10	56	0.65	0.37	0.0264
GO:0048731	System development	204	3867	0.12	0.36	0.0060
GO:0042127	Regulation of cell population proliferation	101	1669	0.18	0.36	0.0093
GO:0016477	Cell migration	62	903	0.23	0.36	0.0116
GO:1901698	Response to nitrogen compound	70	1058	0.22	0.36	0.0116
GO:0006915	Apoptotic process	69	1041	0.22	0.36	0.0121
GO:0010243	Response to organonitrogen compound	65	963	0.23	0.36	0.0121
GO:0110053	Regulation of actin filament organization	26	276	0.37	0.36	0.0182
GO:0006563	L-serine metabolic process	5	12	1.02	0.36	0.0376
GO:0048870	Cell motility	70	1061	0.22	0.35	0.0121
GO:0012501	Programmed cell death	71	1084	0.21	0.35	0.0124
GO:0007275	Multicellular organism development	217	4209	0.11	0.34	0.0098
GO:0010604	Positive regulation of macromolecule metabolic process	187	3533	0.12	0.34	0.0099
GO:0032879	Regulation of localization	121	2103	0.16	0.34	0.0115
GO:0048585	Negative regulation of response to stimulus	97	1612	0.18	0.34	0.0130
GO:0051094	Positive regulation of developmental process	83	1332	0.19	0.34	0.0143
GO:0042594	Response to starvation	21	206	0.4	0.34	0.0264
GO:0009893	Positive regulation of metabolic process	200	3847	0.11	0.33	0.0121

GO:0065009	Regulation of molecular function	166	3085	0.13	0.33	0.0121
GO:0002376	Immune system process	121	2121	0.15	0.33	0.0137
GO:0009888	Tissue development	102	1723	0.17	0.33	0.0143
GO:0097190	Apoptotic signaling pathway	28	318	0.34	0.33	0.0262
GO:0034599	Cellular response to oxidative stress	22	224	0.39	0.33	0.0290
GO:0097193	Intrinsic apoptotic signaling pathway	18	166	0.43	0.33	0.0323
GO:1902236	Negative regulation of endoplasmic reticulum stress-induced intrinsic apoptotic signaling pathway	6	21	0.85	0.33	0.0477
GO:0048513	Animal organ development	172	3246	0.12	0.32	0.0155
GO:0009605	Response to external stimulus	131	2355	0.14	0.32	0.0179
GO:0009968	Negative regulation of signal transduction	78	1252	0.19	0.32	0.0213
GO:0035295	Tube development	59	880	0.22	0.32	0.0238
GO:0001525	Angiogenesis	28	325	0.33	0.32	0.0312
GO:0009636	Response to toxic substance	22	229	0.38	0.32	0.0344
GO:2001234	Negative regulation of apoptotic signaling pathway	22	230	0.38	0.32	0.0355
GO:0072359	Circulatory system development	60	901	0.22	0.31	0.0245
GO:0008285	Negative regulation of cell population proliferation	50	713	0.24	0.31	0.0262
GO:0030334	Regulation of cell migration	61	927	0.21	0.31	0.0264
GO:0001819	Positive regulation of cytokine production	37	482	0.28	0.31	0.0312
GO:0003018	Vascular process in circulatory system	24	262	0.36	0.31	0.0355
GO:0043405	Regulation of MAP kinase activity	19	185	0.41	0.31	0.0390
GO:0071456	Cellular response to hypoxia	15	128	0.46	0.31	0.0451
GO:0019220	Regulation of phosphate metabolic process	85	1405	0.18	0.3	0.0253
GO:0042592	Homeostatic process	85	1406	0.18	0.3	0.0256
GO:0010648	Negative regulation of cell communication	82	1348	0.18	0.3	0.0264
GO:2000026	Regulation of multicellular organismal development	84	1389	0.18	0.3	0.0264
GO:0023057	Negative regulation of signaling	82	1354	0.18	0.3	0.0290
GO:0035239	Tube morphogenesis	47	669	0.24	0.3	0.0329
GO:0001568	Blood vessel development	38	505	0.27	0.3	0.0355
GO:0002683	Negative regulation of immune system process	34	433	0.29	0.3	0.0355
GO:0043406	Positive regulation of MAP kinase activity	14	116	0.48	0.3	0.0498
GO:0002682	Regulation of immune system process	86	1438	0.17	0.29	0.0293
GO:1901700	Response to oxygen-containing compound	91	1547	0.17	0.29	0.0312
GO:0042060	Wound healing	28	336	0.32	0.29	0.0461
GO:0030154	Cell differentiation	181	3507	0.11	0.28	0.0293
GO:0032502	Developmental process	274	5657	0.08	0.28	0.0293
GO:0048856	Anatomical structure development	251	5117	0.09	0.28	0.0293
GO:0048869	Cellular developmental process	182	3530	0.11	0.28	0.0293
GO:0001817	Regulation of cytokine production	50	739	0.23	0.28	0.0451
GO:0010035	Response to inorganic substance	39	532	0.26	0.28	0.0455

GO:0031667	Response to nutrient levels	35	461	0.28	0.28	0.0477
GO:0050789	Regulation of biological process	518	11655	0.04	0.27	0.0289
GO:0050794	Regulation of cellular process	493	11025	0.05	0.27	0.0293
GO:1901701	Cellular response to oxygen-containing compound	66	1057	0.19	0.27	0.0461
GO:0010629	Negative regulation of gene expression	58	899	0.21	0.27	0.0477
GO:0048468	Cell development	98	1719	0.15	0.26	0.0455
GO:0051049	Regulation of transport	100	1763	0.15	0.26	0.0461

Table S8. List of STRING-enriched GO biological process enrichment of downregulated genes at 12 h.

GO-term	Description	Observed gene count	Background gene count	Strength	Signal	False discovery rate
GO:0006260	DNA replication	43	203	1.2	4.39	2.37e-31
GO:0006261	DNA-templated DNA replication	33	137	1.25	4.09	4.57e-25
GO:0006268	DNA unwinding involved in DNA replication	13	22	1.64	2.99	6.93e-13
GO:0033260	Nuclear DNA replication	13	24	1.6	2.89	1.39e-12
GO:0090329	Regulation of DNA-templated DNA replication	17	57	1.34	2.76	2.39e-13
GO:0032508	DNA duplex unwinding	19	89	1.2	2.48	6.27e-13
GO:0006270	DNA replication initiation	12	29	1.49	2.38	1.34e-10
GO:1902969	Mitotic DNA replication	9	12	1.74	2.25	2.11e-09
GO:0006259	DNA metabolic process	61	785	0.76	2.2	3.01e-24
GO:0022616	DNA strand elongation	10	20	1.57	2.16	2.31e-09
GO:0006281	DNA repair	43	497	0.81	2.08	9.49e-18
GO:0006271	DNA strand elongation involved in DNA replication	9	15	1.65	2.08	7.37e-09
GO:0000724	Double-strand break repair via homologous recombination	18	114	1.07	1.94	1.61e-10
GO:0000727	Double-strand break repair via break-induced replication	8	12	1.69	1.9	4.96e-08
GO:1900264	Positive regulation of DNA-directed DNA polymerase activity	8	13	1.66	1.84	7.67e-08
GO:0006974	Cellular response to DNA damage stimulus	51	744	0.71	1.8	1.48e-17
GO:0006310	DNA recombination	23	235	0.86	1.59	5.98e-10
GO:0006302	Double-strand break repair	21	204	0.88	1.56	2.11e-09
GO:0030174	Regulation of DNA-templated DNA replication initiation	7	15	1.54	1.39	4.31e-06
GO:0000278	Mitotic cell cycle	36	631	0.63	1.25	6.95e-10
GO:1902975	Mitotic DNA replication initiation	5	5	1.87	1.2	3.60e-05
GO:0007049	Cell cycle	55	1246	0.51	1.16	1.50e-11

GO:1903047	Mitotic cell cycle process	31	537	0.63	1.16	1.60e-08
GO:0071897	DNA biosynthetic process	12	92	0.98	1.16	5.73e-06
GO:0033554	Cellular response to stress	63	1572	0.47	1.1	9.47e-12
GO:0022402	Cell cycle process	40	835	0.55	1.08	5.33e-09
GO:0000731	DNA synthesis involved in DNA repair	8	41	1.16	1.0	8.57e-05
GO:0031570	DNA integrity checkpoint signaling	12	113	0.9	0.97	3.95e-05
GO:0051276	Chromosome organization	40	968	0.49	0.9	2.74e-07
GO:0006950	Response to stress	96	3358	0.33	0.85	1.27e-10
GO:0090304	Nucleic acid metabolic process	70	2203	0.37	0.85	5.95e-09
GO:1903934	Positive regulation of DNA primase activity	4	5	1.77	0.82	0.00100
GO:0000075	Cell cycle checkpoint signaling	13	157	0.79	0.81	0.00016
GO:0045005	DNA-templated DNA replication maintenance of fidelity	8	54	1.04	0.8	0.00051
GO:0006287	Base-excision repair, gap-filling	5	14	1.42	0.79	0.0010
GO:0000723	Telomere maintenance	10	97	0.88	0.76	0.00052
GO:0009394	2-deoxyribonucleotide metabolic process	7	43	1.08	0.75	0.0010
GO:0010389	Regulation of G2/M transition of mitotic cell cycle	10	99	0.87	0.74	0.00060
GO:0000077	DNA damage checkpoint signaling	10	105	0.85	0.7	0.00089
GO:0044774	Mitotic DNA integrity checkpoint signaling	9	85	0.89	0.7	0.0011
GO:0006139	Nucleobase-containing compound metabolic process	74	2722	0.3	0.68	1.10e-06
GO:0050896	Response to stimulus	164	7835	0.19	0.66	5.98e-10
GO:0051716	Cellular response to stimulus	139	6357	0.21	0.66	1.03e-08
GO:0006725	Cellular aromatic compound metabolic process	76	2936	0.28	0.63	4.87e-06
GO:0046483	Heterocycle metabolic process	75	2891	0.28	0.63	5.63e-06
GO:0009410	Response to xenobiotic stimulus	20	422	0.55	0.62	0.00064
GO:0051052	Regulation of DNA metabolic process	23	541	0.5	0.6	0.00064
GO:1901987	Regulation of cell cycle phase transition	20	431	0.54	0.6	0.00083
GO:0044260	Cellular macromolecule metabolic process	66	2512	0.29	0.59	3.36e-05
GO:1901360	Organic cyclic compound metabolic process	78	3181	0.26	0.57	2.72e-05
GO:0010564	Regulation of cell cycle process	27	716	0.45	0.57	0.00071
GO:0007093	Mitotic cell cycle checkpoint signaling	10	127	0.77	0.57	0.0033
GO:0051726	Regulation of cell cycle	36	1108	0.38	0.56	0.00046
GO:0044773	Mitotic DNA damage checkpoint signaling	8	81	0.86	0.56	0.0048
GO:0007346	Regulation of mitotic cell cycle	21	493	0.5	0.55	0.0014
GO:0010243	Response to organonitrogen compound	32	963	0.39	0.53	0.00100
GO:0000082	G1/S transition of mitotic cell cycle	8	86	0.84	0.53	0.0067
GO:0006284	Base-excision repair	6	43	1.01	0.53	0.0079
GO:0009628	Response to abiotic stimulus	35	1107	0.37	0.52	0.00100
GO:1901698	Response to nitrogen compound	34	1058	0.38	0.52	0.00100
GO:0097529	Myeloid leukocyte migration	10	138	0.73	0.52	0.0056
GO:0006272	Leading strand elongation	3	4	1.74	0.52	0.0126

GO:1901990	Regulation of mitotic cell cycle phase transition	16	332	0.55	0.51	0.0038
GO:0031297	Replication fork processing	6	45	0.99	0.51	0.0096
GO:0009314	Response to radiation	19	444	0.5	0.5	0.0033
GO:0010948	Negative regulation of cell cycle process	14	272	0.58	0.49	0.0058
GO:0044772	Mitotic cell cycle phase transition	11	173	0.67	0.49	0.0068
GO:0034641	Cellular nitrogen compound metabolic process	79	3463	0.23	0.48	0.00035
GO:0009265	2-deoxyribonucleotide biosynthetic process	4	15	1.3	0.48	0.0155
GO:1901700	Response to oxygen-containing compound	43	1547	0.31	0.47	0.0015
GO:0060326	Cell chemotaxis	12	210	0.63	0.47	0.0079
GO:0010165	Response to X-ray	5	31	1.08	0.47	0.0161
GO:2000278	Regulation of DNA biosynthetic process	9	124	0.73	0.46	0.0121
GO:0044818	Mitotic G2/M transition checkpoint	6	50	0.95	0.46	0.0153
GO:0045786	Negative regulation of cell cycle	16	359	0.52	0.45	0.0080
GO:0050900	Leukocyte migration	13	249	0.59	0.45	0.0090
GO:0043085	Positive regulation of catalytic activity	35	1191	0.34	0.44	0.0036
GO:0051338	Regulation of transferase activity	29	912	0.37	0.44	0.0048
GO:0007095	Mitotic G2 DNA damage checkpoint signaling	5	34	1.04	0.43	0.0223
GO:0009211	Pyrimidine deoxyribonucleoside triphosphate metabolic process	3	6	1.57	0.43	0.0255
GO:0071139	Resolution of recombination intermediates	3	6	1.57	0.43	0.0255
GO:0002573	Myeloid leukocyte differentiation	9	134	0.7	0.41	0.0189
GO:0048522	Positive regulation of cellular process	109	5584	0.16	0.4	0.0017
GO:0010033	Response to organic substance	62	2692	0.23	0.4	0.0036
GO:0051347	Positive regulation of transferase activity	21	586	0.42	0.4	0.0113
GO:0051054	Positive regulation of DNA metabolic process	14	304	0.53	0.4	0.0154
GO:1901991	Negative regulation of mitotic cell cycle phase transition	10	167	0.65	0.4	0.0192
GO:0032201	Telomere maintenance via semi-conservative replication	3	7	1.5	0.4	0.0342
GO:0035701	Hematopoietic stem cell migration	3	7	1.5	0.4	0.0342
GO:0050790	Regulation of catalytic activity	56	2370	0.24	0.39	0.0048
GO:0034764	Positive regulation of transmembrane transport	12	236	0.58	0.39	0.0189
GO:0045003	Double-strand break repair via synthesis-dependent strand annealing	3	8	1.44	0.37	0.0441
GO:0048518	Positive regulation of biological process	116	6207	0.14	0.36	0.0048
GO:0045930	Negative regulation of mitotic cell cycle	11	214	0.58	0.35	0.0302
GO:0090068	Positive regulation of cell cycle process	12	251	0.55	0.35	0.0302
GO:0030595	Leukocyte chemotaxis	9	149	0.65	0.35	0.0352
GO:0036297	Interstrand cross-link repair	5	41	0.96	0.35	0.0441
GO:0044093	Positive regulation of molecular function	40	1587	0.27	0.34	0.0175
GO:0014070	Response to organic cyclic compound	26	861	0.35	0.34	0.0206
GO:0010517	Regulation of phospholipase activity	6	66	0.83	0.34	0.0474

GO:0006996	Organelle organization	72	3470	0.19	0.32	0.0149
GO:0007275	Multicellular organism development	83	4209	0.16	0.31	0.0189
GO:0035556	Intracellular signal transduction	38	1518	0.27	0.31	0.0278
GO:0045787	Positive regulation of cell cycle	14	349	0.47	0.3	0.0471
GO:0009987	Cellular process	226	14826	0.05	0.29	0.0139
GO:0008219	Cell death	30	1118	0.3	0.29	0.0418
GO:0065009	Regulation of molecular function	64	3085	0.19	0.28	0.0346
GO:0044238	Primary metabolic process	125	7156	0.11	0.26	0.0336
GO:0042221	Response to chemical	78	4010	0.16	0.26	0.0441

Table S9. List of STRING-enriched GO biological process enrichment of downregulated genes at 24 h.

GO-term	Description	Observed gene count	Background gene count	Strength	Signal	False discovery rate
GO:0006260	DNA replication	44	203	1.05	3.49	2.57e-26
GO:0090329	Regulation of DNA-templated DNA replication	20	57	1.26	2.84	1.58e-14
GO:0006261	DNA-templated DNA replication	30	137	1.06	2.79	1.28e-17
GO:0032392	DNA geometric change	23	95	1.1	2.49	4.71e-14
GO:0071103	DNA conformation change	24	104	1.08	2.48	2.67e-14
GO:0032508	DNA duplex unwinding	22	89	1.11	2.45	1.21e-13
GO:0000278	Mitotic cell cycle	69	631	0.75	2.33	2.57e-26
GO:1903047	Mitotic cell cycle process	60	537	0.76	2.26	2.78e-23
GO:0006268	DNA unwinding involved in DNA replication	12	22	1.45	2.25	3.92e-10
GO:0006259	DNA metabolic process	73	785	0.68	2.04	2.59e-24
GO:0007049	Cell cycle	103	1246	0.63	2.01	1.12e-31
GO:0022402	Cell cycle process	74	835	0.66	1.95	1.37e-23
GO:0006281	DNA repair	51	497	0.73	1.93	6.77e-18
GO:0098813	Nuclear chromosome segregation	31	229	0.85	1.92	2.53e-13
GO:0033260	Nuclear DNA replication	11	24	1.38	1.89	1.28e-08
GO:0007059	Chromosome segregation	35	286	0.8	1.88	6.21e-14
GO:1900264	Positive regulation of DNA-directed DNA polymerase activity	9	13	1.56	1.85	4.64e-08
GO:0000819	Sister chromatid segregation	23	144	0.92	1.81	6.85e-11
GO:0022616	DNA strand elongation	10	20	1.41	1.79	4.64e-08
GO:0006275	Regulation of DNA replication	22	136	0.92	1.76	1.82e-10
GO:0006271	DNA strand elongation involved in DNA replication	9	15	1.49	1.74	1.08e-07
GO:0006270	DNA replication initiation	11	29	1.29	1.72	5.21e-08

GO:0000280	Nuclear division	35	323	0.75	1.68	1.30e-12
GO:0000070	Mitotic sister chromatid segregation	20	122	0.93	1.66	1.54e-09
GO:0000727	Double-strand break repair via break-induced replication	8	12	1.54	1.6	5.16e-07
GO:1902969	Mitotic DNA replication	8	12	1.54	1.6	5.16e-07
GO:0006974	Cellular response to DNA damage stimulus	58	744	0.61	1.55	1.48e-15
GO:0051276	Chromosome organization	69	968	0.57	1.51	4.98e-17
GO:0051301	Cell division	45	527	0.65	1.51	4.00e-13
GO:0006302	Double-strand break repair	25	204	0.8	1.51	1.03e-09
GO:0010564	Regulation of cell cycle process	54	716	0.59	1.45	6.83e-14
GO:0090068	Positive regulation of cell cycle process	27	251	0.75	1.41	1.98e-09
GO:0006310	DNA recombination	26	235	0.76	1.41	2.73e-09
GO:0140014	Mitotic nuclear division	22	175	0.82	1.41	1.26e-08
GO:0000724	Double-strand break repair via homologous recombination	17	114	0.89	1.34	1.69e-07
GO:0051726	Regulation of cell cycle	68	1108	0.5	1.27	8.64e-14
GO:0051321	Meiotic cell cycle	25	250	0.72	1.24	4.30e-08
GO:0051783	Regulation of nuclear division	18	145	0.81	1.19	6.38e-07
GO:0007088	Regulation of mitotic nuclear division	16	118	0.85	1.17	1.54e-06
GO:0030174	Regulation of DNA-templated DNA replication initiation	7	15	1.38	1.17	2.53e-05
GO:0007346	Regulation of mitotic cell cycle	36	493	0.58	1.12	1.57e-08
GO:0044772	Mitotic cell cycle phase transition	19	173	0.76	1.1	1.38e-06
GO:0051983	Regulation of chromosome segregation	16	128	0.81	1.08	4.06e-06
GO:0033554	Cellular response to stress	79	1572	0.42	1.06	6.03e-12
GO:1901990	Regulation of mitotic cell cycle phase transition	27	332	0.63	1.05	4.19e-07
GO:1902975	Mitotic DNA replication initiation	5	5	1.72	1.05	0.00011
GO:1903046	Meiotic cell cycle process	19	188	0.72	1.01	4.28e-06
GO:1901987	Regulation of cell cycle phase transition	31	431	0.57	1.0	4.03e-07
GO:0071897	DNA biosynthetic process	13	92	0.87	1.0	2.21e-05
GO:0000075	Cell cycle checkpoint signaling	17	157	0.75	0.99	9.48e-06
GO:1905818	Regulation of chromosome separation	14	109	0.82	0.98	2.11e-05
GO:0010948	Negative regulation of cell cycle process	23	272	0.64	0.97	3.10e-06
GO:0007093	Mitotic cell cycle checkpoint signaling	15	127	0.79	0.96	2.02e-05
GO:0009394	2-deoxyribonucleotide metabolic process	9	43	1.04	0.94	1.00e-04
GO:0045841	Negative regulation of mitotic metaphase/anaphase transition	8	33	1.1	0.93	0.00015
GO:0140013	Meiotic nuclear division	17	171	0.71	0.9	2.56e-05
GO:0044843	Cell cycle G1/S phase transition	12	88	0.85	0.89	8.49e-05
GO:0033044	Regulation of chromosome organization	21	253	0.63	0.88	1.62e-05
GO:0051784	Negative regulation of nuclear division	9	48	0.99	0.87	0.00020
GO:0007127	Meiosis I	14	126	0.76	0.85	8.85e-05

GO:0007076	Mitotic chromosome condensation	6	17	1.26	0.84	0.00051
GO:1901988	Negative regulation of cell cycle phase transition	19	225	0.64	0.83	4.59e-05
GO:1901991	Negative regulation of mitotic cell cycle phase transition	16	167	0.7	0.82	8.60e-05
GO:0006950	Response to stress	123	3358	0.28	0.81	2.46e-10
GO:1905820	Positive regulation of chromosome separation	7	28	1.11	0.81	0.00052
GO:2001251	Negative regulation of chromosome organization	11	84	0.83	0.79	0.00029
GO:0051052	Regulation of DNA metabolic process	32	541	0.49	0.78	1.32e-05
GO:0033047	Regulation of mitotic sister chromatid segregation	9	55	0.93	0.78	0.00047
GO:0030261	Chromosome condensation	8	42	1.0	0.78	0.00058
GO:0000082	G1/S transition of mitotic cell cycle	11	86	0.82	0.77	0.00035
GO:0007051	Spindle organization	15	160	0.69	0.76	0.00021
GO:0006284	Base-excision repair	8	43	0.99	0.76	0.00066
GO:0007094	Mitotic spindle assembly checkpoint signaling	7	31	1.07	0.76	0.00084
GO:0033043	Regulation of organelle organization	54	1190	0.37	0.75	2.59e-06
GO:0033045	Regulation of sister chromatid segregation	12	105	0.77	0.75	0.00035
GO:2000573	Positive regulation of DNA biosynthetic process	10	73	0.85	0.75	0.00053
GO:0090304	Nucleic acid metabolic process	85	2203	0.3	0.74	1.97e-07
GO:0006996	Organelle organization	120	3470	0.25	0.73	1.75e-08
GO:0030071	Regulation of mitotic metaphase/anaphase transition	11	91	0.8	0.73	0.00053
GO:1903934	Positive regulation of DNA primase activity	4	5	1.62	0.73	0.0019
GO:0030595	Leukocyte chemotaxis	14	149	0.69	0.71	0.00042
GO:0045930	Negative regulation of mitotic cell cycle	17	214	0.62	0.7	0.00033
GO:0000226	Microtubule cytoskeleton organization	30	542	0.46	0.68	0.00010
GO:0009211	Pyrimidine deoxyribonucleoside triphosphate metabolic process	4	6	1.54	0.68	0.0029
GO:0072527	Pyrimidine-containing compound metabolic process	10	82	0.8	0.67	0.0012
GO:0009219	Pyrimidine deoxyribonucleotide metabolic process	6	24	1.11	0.67	0.0022
GO:0036336	Dendritic cell migration	6	25	1.1	0.65	0.0027
GO:0009265	2-deoxyribonucleotide biosynthetic process	5	15	1.24	0.64	0.0034
GO:0051984	Positive regulation of chromosome segregation	6	26	1.08	0.63	0.0032
GO:1903490	Positive regulation of mitotic cytokinesis	4	7	1.47	0.63	0.0042
GO:0006139	Nucleobase-containing compound metabolic process	93	2722	0.25	0.62	7.18e-06
GO:1902850	Microtubule cytoskeleton organization involved in mitosis	12	129	0.68	0.6	0.0019
GO:0000731	DNA synthesis involved in DNA repair	7	41	0.95	0.6	0.0035
GO:0051782	Negative regulation of cell division	5	17	1.18	0.59	0.0051
GO:1901992	Positive regulation of mitotic cell cycle phase transition	10	92	0.75	0.58	0.0029
GO:0000086	G2/M transition of mitotic cell cycle	8	58	0.86	0.58	0.0038
GO:0034508	Centromere complex assembly	6	29	1.03	0.58	0.0048
GO:0046483	Heterocycle metabolic process	95	2891	0.23	0.57	2.56e-05
GO:0006725	Cellular aromatic compound metabolic process	96	2936	0.23	0.57	2.62e-05

GO:0070192	Chromosome organization involved in meiotic cell cycle	9	76	0.79	0.57	0.0037
GO:0006298	Mismatch repair	6	30	1.02	0.57	0.0054
GO:0002407	Dendritic cell chemotaxis	5	18	1.16	0.57	0.0061
GO:1901989	Positive regulation of cell cycle phase transition	11	115	0.7	0.56	0.0033
GO:1901360	Organic cyclic compound metabolic process	101	3181	0.22	0.55	4.29e-05
GO:0044260	Cellular macromolecule metabolic process	84	2512	0.24	0.55	8.10e-05
GO:0007017	Microtubule-based process	36	803	0.37	0.55	0.00060
GO:0007052	Mitotic spindle organization	10	97	0.73	0.55	0.0040
GO:0045132	Meiotic chromosome segregation	10	98	0.72	0.54	0.0043
GO:0010389	Regulation of G2/M transition of mitotic cell cycle	10	99	0.72	0.54	0.0046
GO:0051338	Regulation of transferase activity	39	912	0.35	0.53	0.00067
GO:0060326	Cell chemotaxis	15	210	0.57	0.53	0.0032
GO:0051716	Cellular response to stimulus	172	6357	0.15	0.52	2.24e-05
GO:0050896	Response to stimulus	203	7835	0.13	0.51	1.39e-05
GO:2000278	Regulation of DNA biosynthetic process	11	124	0.66	0.51	0.0054
GO:0032875	Regulation of DNA endoreduplication	3	3	1.72	0.51	0.0126
GO:0010639	Negative regulation of organelle organization	20	351	0.47	0.49	0.0034
GO:0016043	Cellular component organization	149	5436	0.15	0.48	0.00013
GO:0051054	Positive regulation of DNA metabolic process	18	304	0.49	0.48	0.0048
GO:0050900	Leukocyte migration	16	249	0.52	0.48	0.0051
GO:0009410	Response to xenobiotic stimulus	22	422	0.43	0.46	0.0046
GO:0009987	Cellular process	327	14826	0.06	0.45	2.62e-05
GO:0140694	Non-membrane-bounded organelle assembly	18	314	0.47	0.45	0.0068
GO:0031570	DNA integrity checkpoint signaling	10	113	0.66	0.45	0.0115
GO:0045005	DNA-templated DNA replication maintenance of fidelity	7	54	0.83	0.45	0.0142
GO:0006272	Leading strand elongation	3	4	1.59	0.45	0.0206
GO:0071840	Cellular component organization or biogenesis	150	5639	0.14	0.44	0.00055
GO:1904029	Regulation of cyclin-dependent protein kinase activity	10	114	0.66	0.44	0.0123
GO:0032465	Regulation of cytokinesis	9	94	0.7	0.44	0.0142
GO:0034641	Cellular nitrogen compound metabolic process	101	3463	0.18	0.43	0.0012
GO:0050790	Regulation of catalytic activity	75	2370	0.22	0.43	0.0017
GO:0071674	Mononuclear cell migration	10	116	0.65	0.43	0.0138
GO:0007131	Reciprocal meiotic recombination	7	56	0.81	0.43	0.0172
GO:0051231	Spindle elongation	4	13	1.2	0.43	0.0226
GO:0090235	Regulation of metaphase plate congression	4	13	1.2	0.43	0.0226
GO:0051225	Spindle assembly	9	96	0.69	0.42	0.0162
GO:0000723	Telomere maintenance	9	97	0.68	0.42	0.0172
GO:0006287	Base-excision repair, gap-filling	4	14	1.17	0.41	0.0279
GO:1905821	Positive regulation of chromosome condensation	3	5	1.49	0.41	0.0310
GO:0035556	Intracellular signal transduction	52	1518	0.25	0.4	0.0054

GO:0009264	Deoxyribonucleotide catabolic process	5	28	0.97	0.39	0.0314
GO:0046386	Deoxyribose phosphate catabolic process	5	28	0.97	0.39	0.0314
GO:0051128	Regulation of cellular component organization	72	2365	0.2	0.37	0.0071
GO:0065004	protein-DNA complex assembly	14	229	0.5	0.37	0.0211
GO:0000281	Mitotic cytokinesis	8	84	0.69	0.37	0.0310
GO:0031297	Replication fork processing	6	45	0.84	0.37	0.0329
GO:0010032	Meiotic chromosome condensation	3	6	1.41	0.37	0.0430
GO:0044774	Mitotic DNA integrity checkpoint signaling	8	85	0.69	0.36	0.0327
GO:0010517	Regulation of phospholipase activity	7	66	0.74	0.35	0.0381
GO:0000076	DNA replication checkpoint signaling	4	17	1.09	0.35	0.0476
GO:0071824	protein-DNA complex subunit organization	15	268	0.46	0.34	0.0302
GO:0042770	Signal transduction in response to DNA damage	10	135	0.59	0.34	0.0373
GO:0000079	Regulation of cyclin-dependent protein serine/threonine kinase activity	9	111	0.62	0.34	0.0385
GO:0051302	Regulation of cell division	12	188	0.52	0.33	0.0373
GO:0097529	Myeloid leukocyte migration	10	138	0.58	0.32	0.0430
GO:0007010	Cytoskeleton organization	42	1229	0.25	0.31	0.0270
GO:0065009	Regulation of molecular function	86	3085	0.16	0.3	0.0226
GO:0009314	Response to radiation	20	444	0.37	0.29	0.0467
